# Comparative transcriptome analysis reveals ecological adaption of cold tolerance in northward invasion of *Alternanthera philoxeroides*

**DOI:** 10.1186/s12864-020-06941-z

**Published:** 2020-08-02

**Authors:** Landi Luo, Xiangxiang Kong, Zean Gao, Yan Zheng, Yunqiang Yang, Xiong Li, Danni Yang, Yupeng Geng, Yongping Yang

**Affiliations:** 1 School of Ecology and Environmental Science, Institute of Ecology and Geobotany, Yunnan University, Kunming, 650504 China; 2 Key Laboratory for Plant Diversity and Biogeography of East Asia, Kunming Institute of Botany, Chinese Academy of Sciences, Kunming, 650201 China; 3 Plant Germplasm and Genomics Center, Kunming Institute of Botany, Chinese Academy of Sciences, Kunming, 650201 China; 4 University of Chinese Academy of Sciences, Beijing, 100049 China

**Keywords:** Cold resistance, RNA-Seq, Epigenetic regulation, *Alternanthera philoxeroides*, Plant invasion

## Abstract

**Background:**

*Alternanthera philoxeroides* (alligator weed) is a highly invasive alien plant that has continuously and successfully expanded from the tropical to the temperate regions of China via asexual reproduction. During this process, the continuous decrease in temperature has been a key limiting environmental factor.

**Results:**

In this study, we provide a comprehensive analysis of the cold tolerance of alligator weed via transcriptomics. The transcriptomic differences between the southernmost population and the northernmost population of China were compared at different time points of cold treatments. GO enrichment and KEGG pathway analyses showed that the alligator weed transcriptional response to cold stress is associated with genes encoding protein kinases, transcription factors, plant-pathogen interactions, plant hormone signal transduction and metabolic processes. Although members of the same gene family were often expressed in both populations, the levels of gene expression between them varied. Further ChIP experiments indicated that histone epigenetic modification changes at the candidate transcription factor gene loci are accompanied by differences in gene expression in response to cold, without variation in the coding sequences of these genes in these two populations. These results suggest that histone changes may contribute to the cold-responsive gene expression divergence between these two populations to provide the most beneficial response to chilling stimuli.

**Conclusion:**

We demonstrated that the major alterations in gene expression levels belonging to the main cold-resistance response processes may be responsible for the divergence in the cold resistance of these two populations. During this process, histone modifications in cold-responsive genes have the potential to drive the major alterations in cold adaption necessary for the northward expansion of alligator weed.

## Background

*Alternanthera philoxeroides* (alligator weed), which originated in South America, has invaded many countries [1]. This species is an invasive plant in China, causing economic losses and ecological damage [2]. *A. philoxeroides* is a perennial herb that can occasionally reproduce sexually in its native areas, but only asexually in invaded areas, including the United States, China, Australia, etc. [3, 4]. Previous studies have shown that small underground stems or storage roots can regenerate into new plants via asexual propagation [5, 6], which results in a very consistent genetic background in this weed [7, 8]. Despite its low level of DNA sequence variation, alligator weed can adapt to diverse habitats [9, 10]. This particular reproductive pattern allows for adaptation to climate and environmental variations at geographical scales. As a plant that thrives in humid environments, originating in the tropics, *A. philoxeroides* was originally distributed in the humid and semi-humid areas around the southern Yellow River in China. Since then, this weed has gradually spread northward to Jinan, Shandong Province, where it has to endure low winter temperatures to survive. Surprisingly, alligator weed is far more tolerant to low temperature than its specific natural enemy, *Diptera pectoralis*, populations of which are significantly reduced at high latitudes [11]. Therefore, the cold tolerance of *A. philoxeroides* is crucial for its northward invasion via asexual propagation.

Low temperature is a major environmental factor that limits the geographical distribution of plants and plant growth [12]. Cold tolerance adaption, especially freezing tolerance adaption substantially influences the successful invasion and expansion of alien plants [13]. Plants have evolved various mechanisms in the physiological and biochemical processes to adapt to cold stress [14]. Epigenetic regulation, including histone modification, DNA methylation and noncoding RNAs, can regulate gene expression and produce inheritable phenotypic variation without altering DNA sequences, thus playing an important role in plant responses to cold stress and cold tolerance evolution [15].

For asexually reproducing species, the role of the evolutionary adaptability of epigenetic modifications may be of particular concern [16]. Many invasive plants rely on asexual reproduction to spread. Despite their low level of genetic variation, they can adapt to a wide geographical distribution and highly heterogeneous environments [17]. Epigenetic modification may play an important role during this process. Epigenetic variation can significantly affect the abiotic stress tolerance and geographic expansion of plants, enabling long-term natural selection [14, 18]. Xie et al. suggested that *ICE1* demethylation primarily determines the invasion of crofton weed (*Ageratina adenophora*) in the north of China, and *ICE1* demethylation has also been responsible for the phenotypic divergence in freezing stress tolerance in *Arabidopsis thaliana* [19, 20]. Niche modelling analysis showed that the suitability of habitats for *A. philoxeroides* decreased gradually from south to north in eastern China, and temperature was the main factor affecting this suitability [8]. However, the genetic and molecular mechanisms of cold tolerance divergence, which include the ability for strong radiation of the population and expansion into the cold environment of *A. philoxeroides* remain largely unclear.

In this study, we provide a comprehensive view of the molecular mechanisms in the cold resistance divergence of alligator weeds between the southernmost population and the northernmost population of China based on comparative transcriptome analyses. The major cold-responsive processes in *A. philoxeroides* were summarised, and the epigenetic diversity of several candidate cold-resistant genes was studied between these two populations. The differentiation of histone H3K4mec3 and H3K9ac modifications at the candidate transcription factor loci suggests that epigenetic regulation may play a role in the enhanced cold resistance of *A. philoxeroides* during its northward expansion.

## Results

### Differential responses of two alligator weed populations to freezing stress

**Fig. 1:**
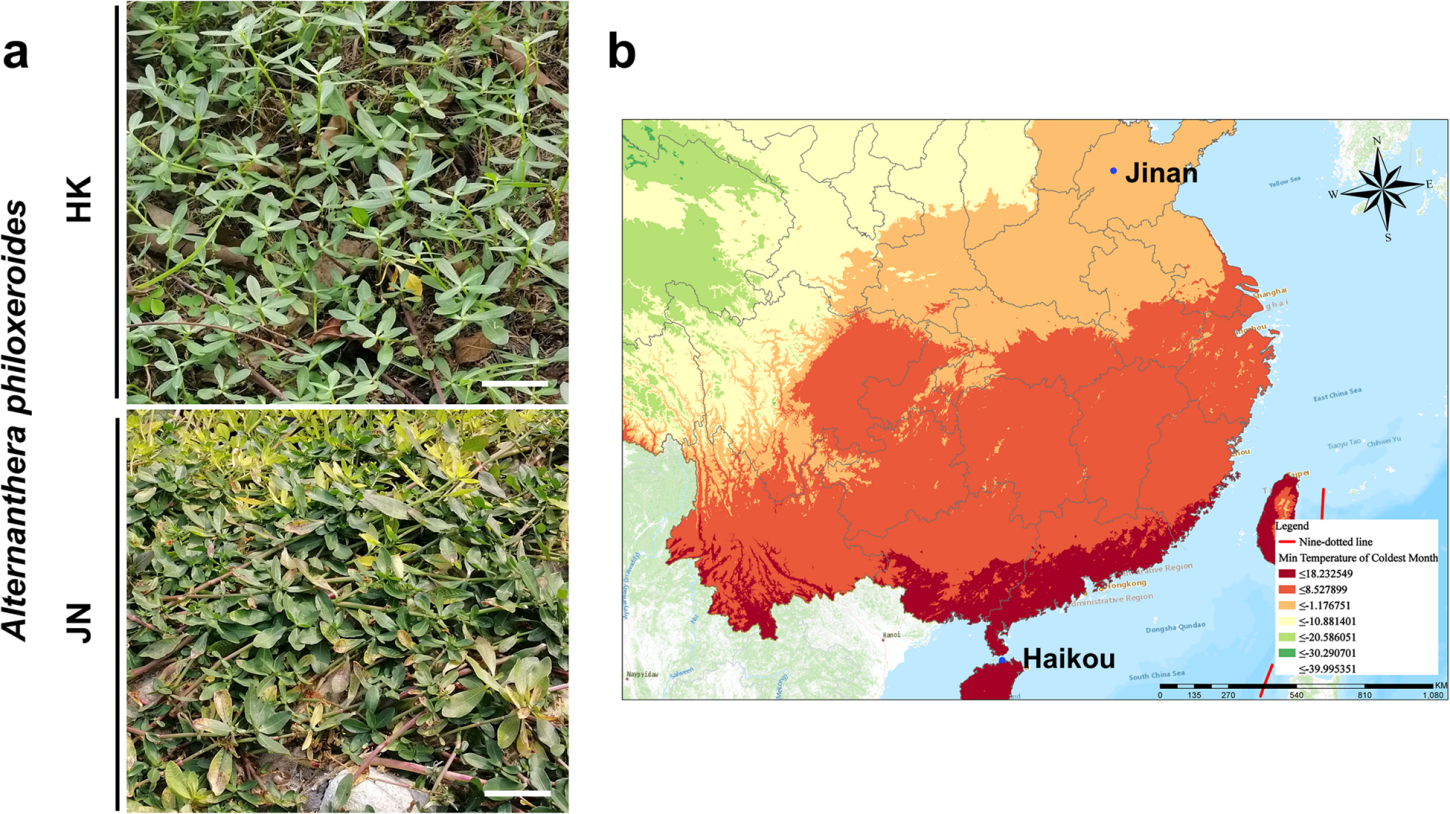
*Alternanthera philoxeroides* in Haikou city (HK), Hainan Province and Jinan city (JN), Shandong Province, China. **a**. Morphology of *Alternanthera philoxeroides* at the collection site of the HK and JN populations (bar = 5 cm). **b**. Geographical distribution of the HK and JN populations in China. The map was created using ArcGIS Pro software (version 2.3.0) (https://www.esri.com/zh-cn/arcgis/products/arcgis-pro/overview)

**Fig. 2:**
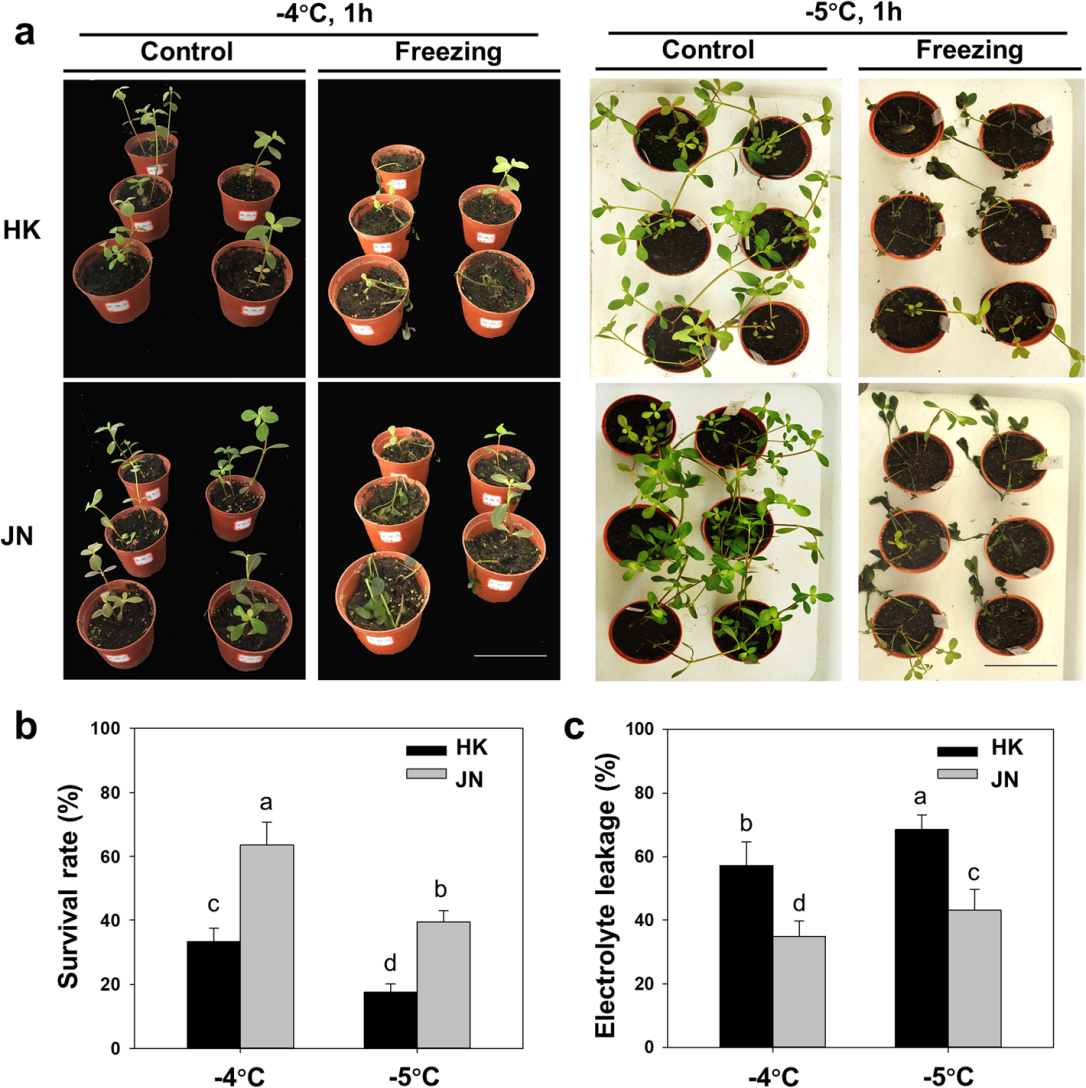
Comparison of cold resistance among the HK and JN populations of *A. philoxeroides*. **a**. Representative phenotypes of the HK and JN plants under freezing stress (bar = 9 cm). **b**. Survival rates of HK and JN plants after freezing stress. **c**. Electrolyte leakage (EL) of the leaves of HK and JN plants after freezing stress

**Fig. 3:**
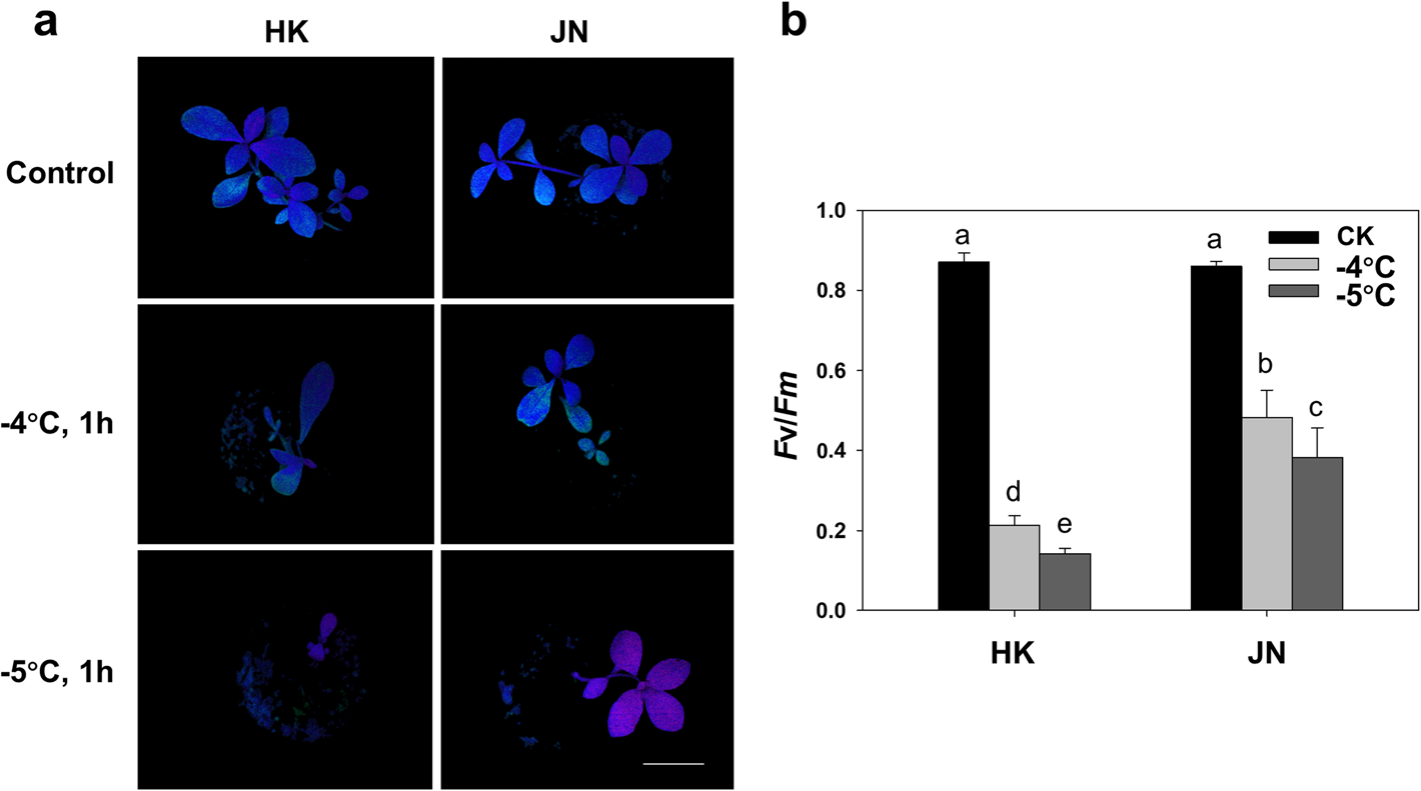
Chl fluorescence imaging and *Fv*/*Fm* ratios of HK and JN plants after freezing stress. **a**. Chl fluorescence imaging of HK and JN plants after freezing stress (bar = 2 cm). **b**. *Fv*/*Fm* ratios of HK and JN plants after freezing stress

Alligator weed has invaded diverse climatic regions from the Hainan (tropical) to Shandong (temperate zone) areas by asexual reproduction [8]. In this study, we collected the southernmost population from Haikou city of China (termed HK) and the northernmost population from Jinan city of China (termed JN), which showed different morphology of upright growth in HK and creeping growth in JN (Fig. 1). We then compared their cold tolerance using transplanted seedlings derived from local vegetative populations. We first analysed the responses of the seedlings to freezing temperatures. When the alligator weed was subjected to freezing treatments of − 4 °C and − 5 °C for 1 h, both HK and JN populations suffered freeze injury. We found that the JN population showed strong resistance to cold stress, whereas the HK population exhibited increased sensitivity to freezing treatments (Fig. 2a). We further analysed the survival rates after cold stress. We observed that the JN plants had higher survival rates than the HK plants after recovery under warm conditions (Fig. 2b). Consistent with these phenotypes, the relative electrolyte leakage levels in HK plants were obviously higher compared to those in the JN plants after freezing (Fig. 2c). The Chl fluorescence of the HK population was prominently repressed following freezing stress, with a lower *Fv/Fm* rate than that of the JN population (Fig. 3). These results indicate that the JN population had stronger cold tolerance than the HN population when exposed to freezing temperatures. The phenotypic differences in cold tolerance between the HK and JN populations may be mechanism-based.

### Transcriptomic analyses of alligator weed plants following cold stress

To further explore the mechanisms underlying the cold-response strategies of *A. philoxeroides*, we performed transcriptomic analyses by deep RNA-Seq of the HK and JN populations following different low-temperature treatments (4 °C for 0, 12, 24 h). Eighteen RNA libraries were constructed with three biological replicates at each point for both HK and JN populations. A total of 830 million clean reads were obtained from these libraries after removal of adaptor sequences, low-quality reads and reads with greater than 5% ambiguous N bases, and approximately 43.54–50.85 million reads were generated per library with 89.00–90.25% clean reads of Q30 (Additional file 2: Table S1). Using the Trinity program to assemble the clean reads and using the TransDecoder software to identify candidate coding regions in Unigene, a total of 195,260 unigenes and 117,423 candidate coding regions of the unigenes were generated after clustering (Additional file 3: Protein.fa). The N50 was 1391 bp, and the GC content was 39.57% (Additional file 4: Table S2). The mean size of these unigenes was 901 bp, and 90.87% of these unigenes were < 2500 bp in length (Additional file 1: Fig. S1). Quality assessment of assembled transcripts using BUSCO software showed high quality and accuracy of transcriptome assembly (Additional file 1: Fig. S2; Additional file 5: Table S3). A principal component analysis (PCA) carried out by the principop function in R software showed that replicates of HK and JN independent samples before and after cold treatments clustered closely (Additional file 1: Fig. S3). These data indicated that the assembly was considered high quality and could be used in further analyses.

### Annotation of unigenes of *A. philoxeroides*

For identification of the putative function of these acquired unigenes, the assembled unigenes were annotated against seven functional databases, including NR, NT, Swiss-Prot, KEGG, KOG, Pfam and GO (E-value < 1.0 × 10^− 5^). In total, 143,751 unigenes were annotated in at least one of the abovementioned databases (Table 1). Among these databases, the NR database successfully annotated the most unigenes (70.61%), followed by 59.24% in the NT database, 56.10% in KOG, and 55.83% in KEGG. The unigene sequences were matched to the known gene sequences mainly from *Beta vulgaris subsp. vulgaris* (37.67%), *Chenopodium quinoa* (31.87%) and *Spinacia oleracea* (19.74%) in the NR database (Additional file 1: Fig. S4).

**Table 1:** Annotation statistics of unigenes of *A. philoxeroides*

Values	Number	Percentage
Total	195,260	100%
NR	137,870	70.61%
NT	115,664	59.24%
Swiss-Prot	104,960	53.75%
KEGG	109,015	55.83%
KOG	109,546	56.10%
Pfam	107,314	54.96%
GO	31,927	16.35%
Intersection	17,690	9.06%
Overall	143,751	73.62%

### Comparative transcriptome changes in the HK and JN populations in response to cold stress

**Fig. 4:**
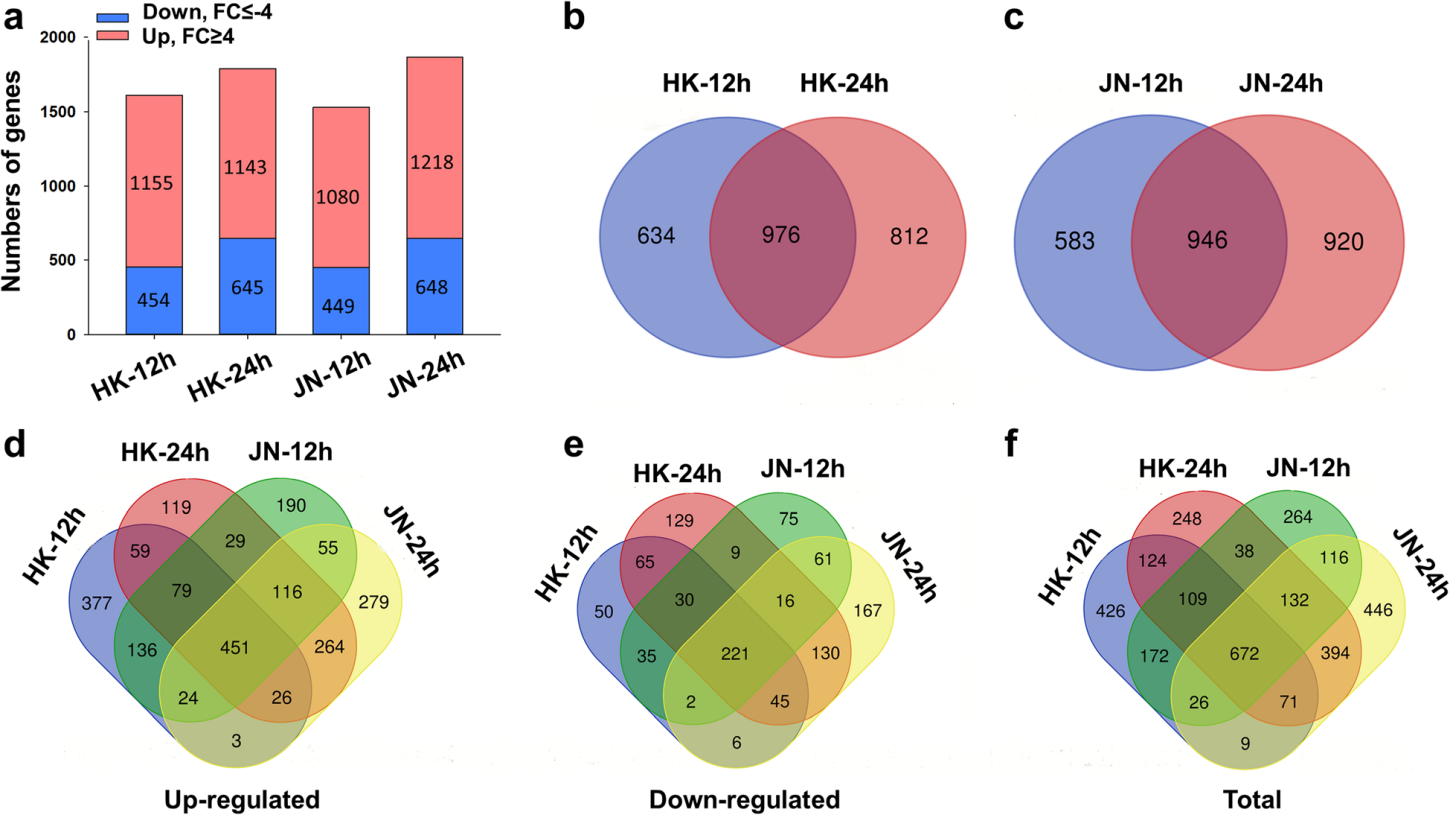
Comparative analysis of gene expression of DEGs between the HK and JN populations under cold stress. **a**. Numbers of upregulated and downregulated genes in the HK and JN populations at different time points of cold treatment. The vertical axis represents the numbers of upregulated and downregulated genes at different time points in HK and JN. *Q* ≤ 0.05, Fold Change (FC): |Log_2_(fold-change)| ≥4. **b**-**c**. Venn diagram analysis showing the common and exclusive DEGs at different time points of cold treatment in HK or JN. **d**-**f**. Cross-comparison Venn diagram showing the common and unique DEGs following cold treatments in the HK and JN populations

To reveal relationships between the differences in cold tolerance and gene expression in the HK and JN populations, we compared their different gene expression patterns using DESeq (Q ≤ 0.05), with the gene expression levels presented as FPKM values. A total of 3247 differentially expressed genes (DEGs) were identified after different durations of cold treatment with a threshold of |Log_2_(fold-change)| ≥ 4 (*Q*-value < 0.01) in HK and JN plants compared to the plants without cold stress (Additional file 6: Table S4). Compared with that in plants with no cold treatment, the number of upregulated genes was much higher than that of downregulated genes in both HK and JN cold-treated plants. Among these 3247 DEGs, 1155 were upregulated and 454 were downregulated in HK after 12 h at 4 °C, 1143 were upregulated and 645 were downregulated in HK after 24 h at 4 °C, 1080 were upregulated and 449 were downregulated in JN after 12 h at 4 °C, and 1218 were upregulated and 648 were downregulated in JN after 24 h at 4 °C (Fig. 4a, Additional file 7: Table S5).

**Fig. 5:**
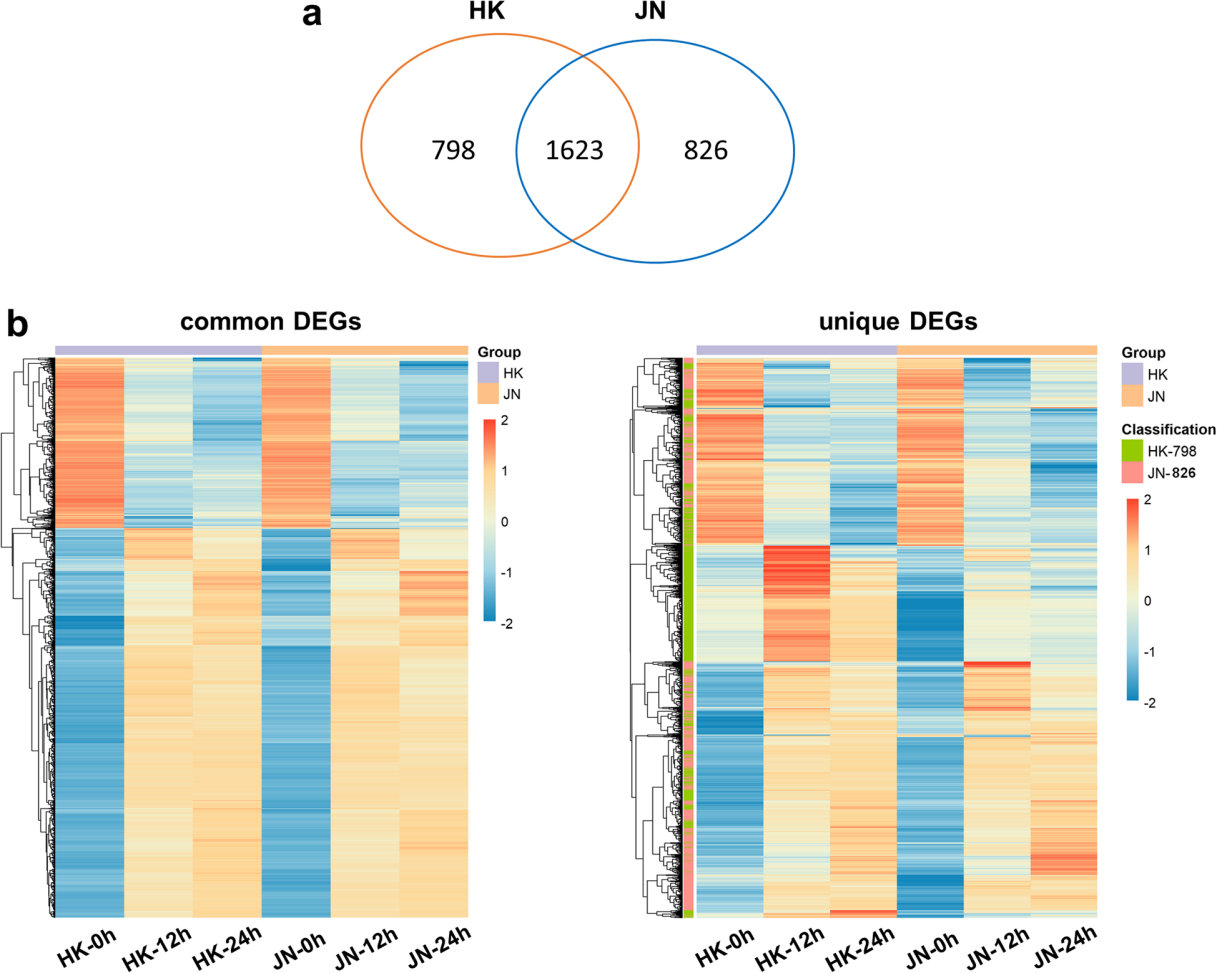
Hierarchical cluster analysis of the expression patterns of DEGs between the HN and JN populations. **a**. Venn diagram analysis showing the common and unique DEGs in HK and JN. **b**. Hierarchical cluster analysis of the expression patterns between the common and unique DEGs in HK and JN

We further compared the DEGs during the two cold treatment time points in HK and JN and found a large overlap of DEGs across the two time points (12 h and 24 h at 4 °C) in HK (976 unigenes) and JN (946 unigenes) (Fig. 4b-c). In addition, the commonality of genes upregulated or downregulated was also analysed between the HK and JN populations at 12 and 24 h of cold treatment (Fig. 4d-e). Comparative analysis revealed that a relatively large overlap in upregulated DEGs (1128 sequences) was found in HK (67%) and JN (68.3%). Analogously, a large number of downregulated genes (494 sequences) were divided into the common datasets (i.e., 66.9% in HK and 62.1% in JN). A similar comparison of the total DEGs across the four samples showed extensive overlap between HK and JN during cold stress, which had 1623 common DEGs (Fig. 4f). Hierarchical clustering analysis was performed on the expression patterns of the 1623 common previously described DEGs and 1624 unique DEGs in HK and JN that were identified following the cold treatments (Fig. 5a-b, Additional file 8: Table S6). The expression patterns of the DEGs were similar in both HK and JN populations, but their expression levels were different.

### GO enrichment analysis of DEGs

**Fig. 6:**
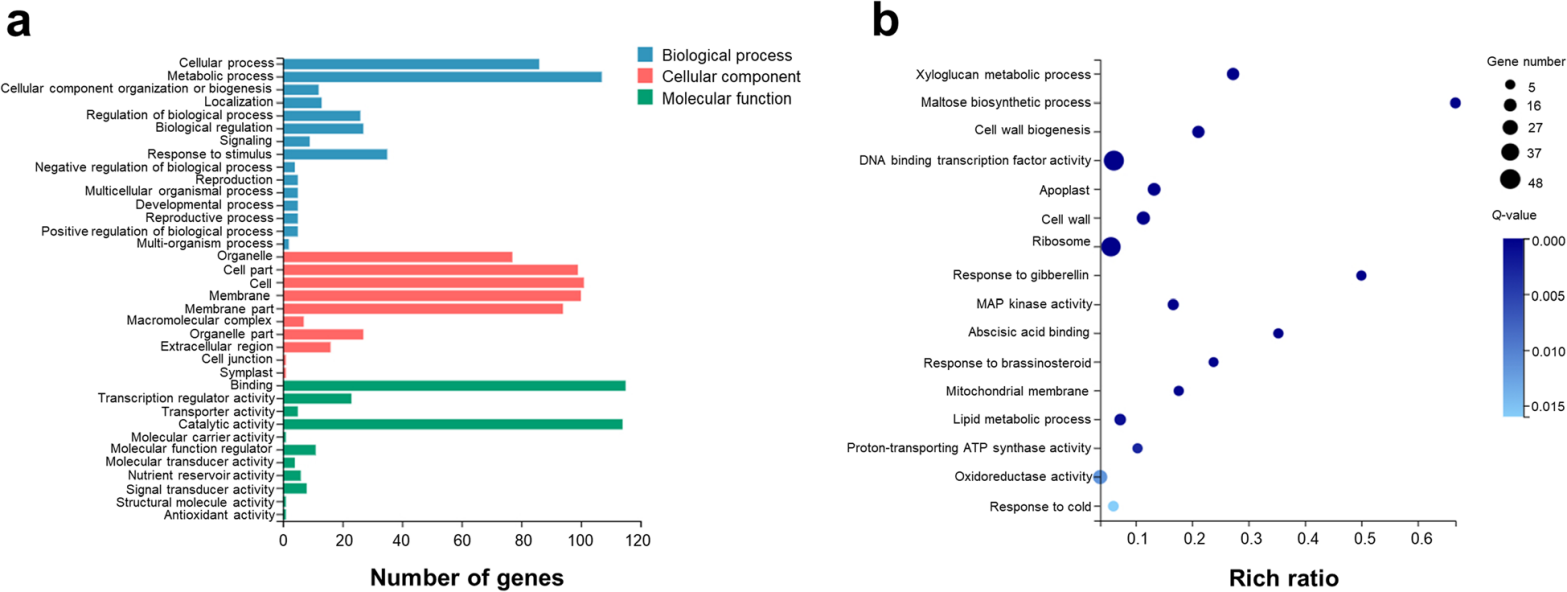
GO annotation and enrichment analysis. **a**. GO annotation results for the total number of DEGs (Fisher’s exact test, *Q*-value≤0.05). **b**. GO enrichment analysis. The top sixteen pathways with minimum *Q values* (*Q*-value≤0.05) are shown

The 3247 significantly DEGs were further categorised into three major categories: 16 biological processes, 13 cellular components, and 11 molecular functions (Fig. 6a, Additional file 9: Table S7). Genes associated with metabolic process, cellular process and response to stimulus were the major abundant functional groups in the biological processes category. Based on GO enrichment analysis (Fisher’s exact test, *Q*-value≤0.05), xyloglucan metabolic process (GO:0010411), maltose biosynthetic process (GO:0000024), lipid metabolic process (GO:0006629) and cell wall biogenesis (GO:0042546) were significantly enriched in the metabolic process category after the cold treatments (Fig. 6b, Additional file 10: Table S8). Genes related to response to cold (GO:0009409), response to gibberellin (GO:0009739), response to brassinosteroid (GO:0009741), and abscisic acid binding (GO:0010427) were mainly involved in response to stimulus processes. The cellular process category included enriched terms such as ribosome (GO:0005840), cell wall (GO:0005618), mitochondrial membrane (GO:0031966) and apoplast (GO:0048046), which belong to cell part, cell and membrane groups. In the molecular function category, binging, catalytic activity, transcription regulator activity and structural molecule activity were the key processes involved in the response to cold stress. The major subcategories were MAP kinase activity (GO:0004707), proton-transporting ATP synthase activity (GO:0046933), DNA binding transcription factor activity (GO:0003700) and oxidoreductase activity (GO:0016491).

### KEGG pathway enrichment analysis of cold stress response DEGs

**Fig. 7:**
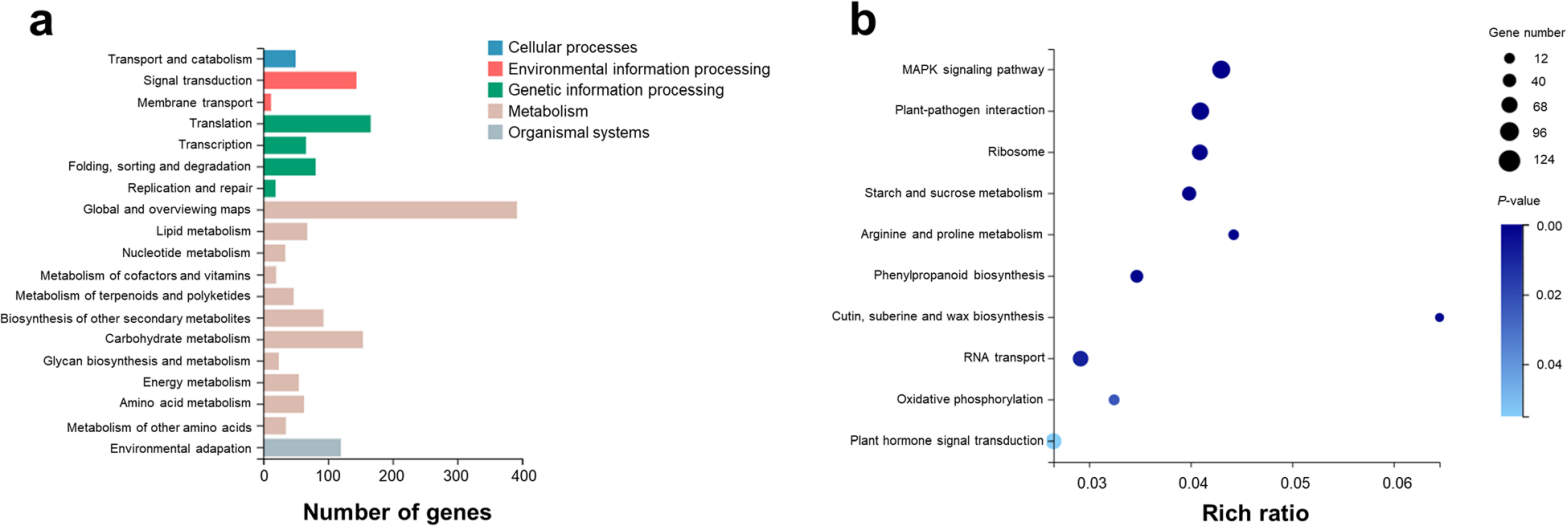
KEGG annotation and enrichment analysis. **a**. KEGG pathway annotation for the total number of DEGs (*Q*-value≤0.05). **b**. KEGG enrichment analysis. The top ten pathways with minimum *Q values* (*Q*-value≤0.05) are shown

According to the results of KEGG annotation and enrichment analysis, the significantly DEGs were classified into various biological pathways. DEGs from both the HK and JN populations after cold stress were categorised into 19 KEGG pathways (*Q*-value≤0.05) (Fig. 7a). DEGs from the HK and JN populations were assigned to the same 19 significantly enriched pathways (Additional file 1: Fig. S5). The major pathways included signal transduction (204), translation (203), carbohydrate metabolism (195), environmental adaptation (163) and folding, sorting and degradation (123) pathways (Additional file 11: Table S9). KEGG enrichment analysis according to the minimum *Q* value and the candidate gene number showed that transcripts involved in the MAPK signalling pathway (ko04016), plant-pathogen interaction (ko04626), ribosome (ko03010), starch and sucrose metabolism (ko00500), phenylpropanoid biosynthesis (ko00940), arginine and proline metabolism (ko00330), RNA transport (ko03013), oxidative phosphorylation (ko00190) and plant hormone signal transduction (ko04075) appeared to be the most important pathways in the alligator weed response to low temperatures (Fig. 7b, Additional file 1: Fig. S6, Additional file 12: Table S10). Comparative analysis of KEGG enrichment during the two cold treatment time points in HK and JN revealed that genes related to the MAPK signalling pathway, plant-pathogen interaction, RNA transport, oxidative phosphorylation and plant hormone signal transduction were quickly activated at the initial 12 h of cold treatment in both HK and JN populations following changes in related DEGs numbers (Additional file 1: Fig. S7, Additional file 13: Table S11). In contrast, the processes of secondary metabolism and biosynthesis were specifically enriched at the later 24 h point in both populations. These analyses indicated that the pathways in response to cold were common in both populations, but varied significantly between the early and late time points in *A. philoxeroides*.

### Genes potentially involved in cold tolerance in *A. philoxeroides*

**Fig. 8:**
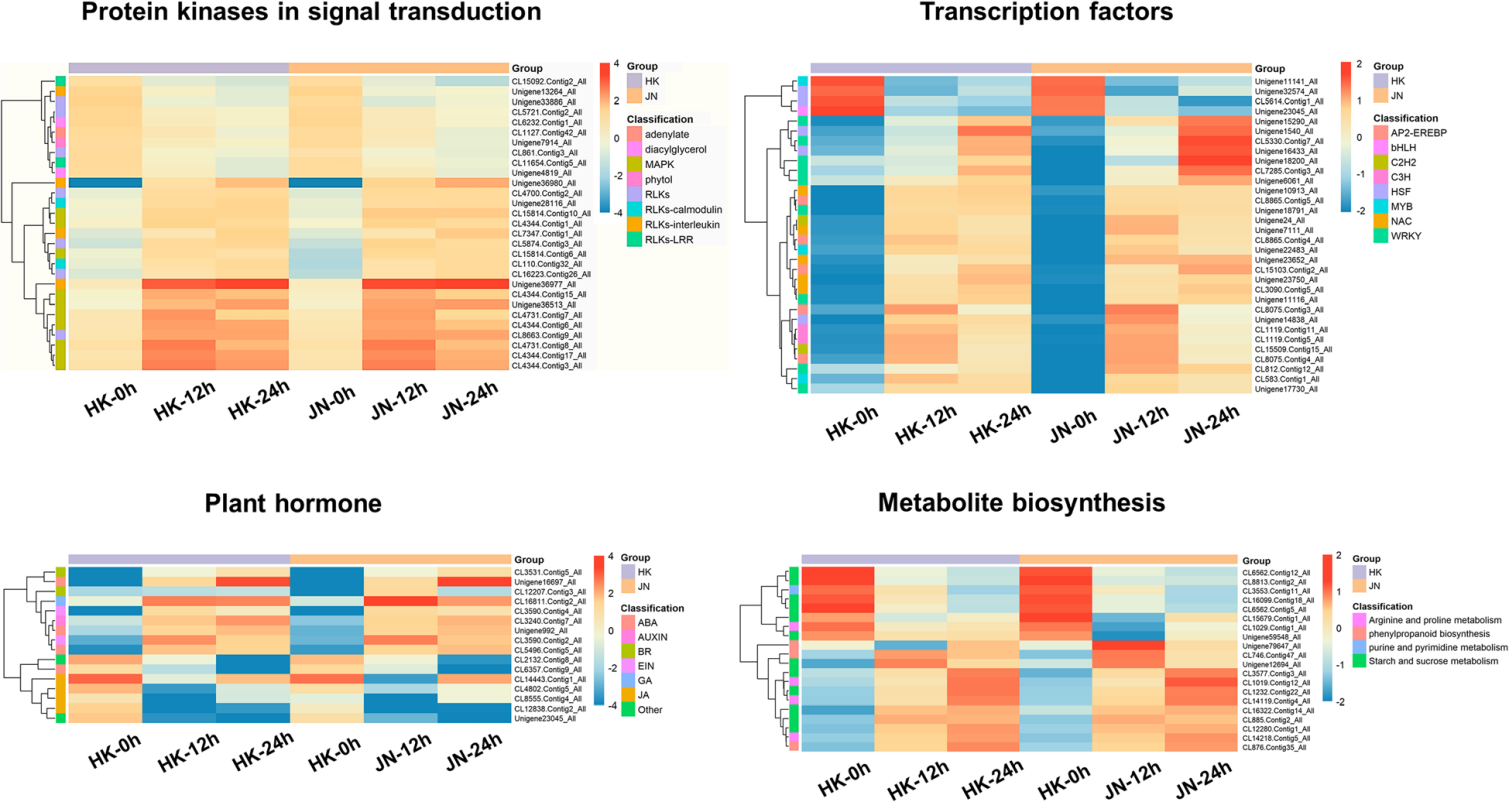
Heatmap analysis of significantly enriched pathways potentially related to cold resistance in *A. philoxeroides*

Based on the KEGG pathway and GO functional enrichment analyses, multiple resistance-related genes were identified as having differential transcriptional responses during cold stress. Most of these genes belong to protein kinases involved in signal transduction, transcription factors, plant hormones, and metabolite biosynthesis groups (Fig. 8). The gene families potentially responsible for cold tolerance in *A. philoxeroides* are discussed further below.

### Identification of protein kinase-related genes

Signal transduction is well documented in plants responding to both abiotic and biotic stresses. Protein kinases play important roles in signal transduction. The MAPK signalling pathway exhibited the most DEGs with minimum *Q* values, indicating that MAPK protein kinases play significant roles during the adaptation of alligator weed to cold stress (Additional file 14: Table S12). Among these identified genes, 23 genes were involved in mitogen-activated protein kinase (MAPK) cascades, including *MAPKs*, *MAPKKs* and *MAPKKKs*, such as *MAPK3/4/6*, *MAPKK9* and *MAPKKK17/18*. All of these MAPK kinases were significantly increased in response to cold stress. The receptor-like kinases (RLKs) are the most key kinases in triggering stress resistance responses. Our DEG dataset revealed 139 genes predicted to encode kinases with different transcriptional levels between HK and JN plants under low temperature. More than 37 receptor-like kinase genes were identified, some of which, including LRR receptor-like serine/threonine-protein kinase, interleukin-1 receptor-associated kinase and calmodulin-binding receptor-like cytoplasmic kinase, were upregulated in both HK and JN plants during cold stress. However, a majority of these upregulated RLK genes in JN had significantly higher expression levels than those in HK, indicating that the JN population had higher kinase activity than the HK population under cold stress. In contrast, a number of kinases, such as adenylate kinase, phytol kinase and diacylglycerol kinase, were downregulated by cold stress. These genes encoding protein kinases participate in several pathways, including signal transduction, plant-pathogen interactions and hormone signalling during abiotic stress.

### Identification of transcription factor-related genes

Transcription factors are important functional regulators in abiotic stress responses. In cold stress, transcription factors regulate upstream cold signals to activate downstream genes with cold tolerance. Based on our DEG data, a total of 252 transcription factors (TFs) were identified, including the WRKY, Tify, zinc finger, NAC, MYB, bHLH, GRAS and AP2-EREBP families (Additional file 15: Table S13). Among these families, 39 *WRKYs* were identified. *WRKY28*, *WRKY40*, *WRKY18*, *WRKY62* and *WRKY60* were strongly upregulated in both HK and JN during cold stress, some of which had higher transcriptional levels in JN than in HK. Other *WRKYs,* such as *WRKY8*, *WRKY30* and *WRKY76,* also significantly increased, though at a lower level. Among the 30 zinc finger genes containing the C2H2 and C3H domains, their expression levels were stable at each cold treatment time point in HK and JN. All 22 *NACs* that were identified were upregulated after cold treatment, with 12 *NACs* in JN increasing more than those in HK. In addition, two heat stress transcription factors and ethylene-responsive transcription factors, which are involved in plant hormone signalling, were also identified in our DEG data, with distinct expression patterns in the HK and JN populations.

### Plant hormone signalling related genes

Plant hormone signalling pathways have important functions in the plant response to abiotic stress. We identified genes involved in various phytohormone signalling pathways in cold stress (Additional file 16: Table S14). Four genes encoding jasmonate ZIM domain-containing protein (*TIFY5A*, *TIFY9*, *TIFY10A* and *TIFY10B*), as repressors of JA signalling, were more highly repressed in HK than in JN. In addition, abscisic acid (ABA) signalling genes that function as abscisic acid receptors (*PYL9*, *PYL8*, *PYL4*) were characterised, and these DEGs were significantly activated during the cold treatments, with higher expression levels in JN at the 24 h time point than at the 12 h time point. The ABA coreceptor phosphatase 2C (PP2C) gene (*protein phosphatase 2C 37-like*) was suppressed under cold stress. Additionally, ethylene and auxin signal pathway genes, such as *ethylene-responsive transcription factor 1* and *auxin-induced protein 22D*, were upregulated in both HK and JN under all low temperature conditions. These results suggested that genes involved in plant hormone signalling composed a regulatory network in response to cold stress in *A. philoxeroides*.

### Metabolite biosynthesis

Cold stress influences diverse groups of metabolic biosynthesis processes. Our GO and KEGG pathway enrichment analyses identified multiple metabolite synthesis-related DEGs that were associated with starch and sucrose metabolism, arginine and proline metabolism, phenylpropanoid biosynthesis and purine and pyrimidine metabolism (Additional file 17: Table S15). Under cold stress, the expression of these DEGs related to amylase, glucosidase, fructofuranosidase, fructokinase and sucrose-phosphate synthase was upregulated, while glucose-1-phosphate adenylyltransferase, 1,4-alpha-glucan branching enzyme and trehalose 6-phosphate phosphatase were downregulated in both JN and HK. Most genes associated with arginine and proline metabolism were evidently upregulated in response to cold stress, including polyamine oxidase, arginine decarboxylase and proline dehydrogenase, indicating that polyamine and proline play an important role in the alligator weed response to low temperature.

### Quantitative real-time PCR validation of RNA-Seq-based DEGs

**Fig. 9:**
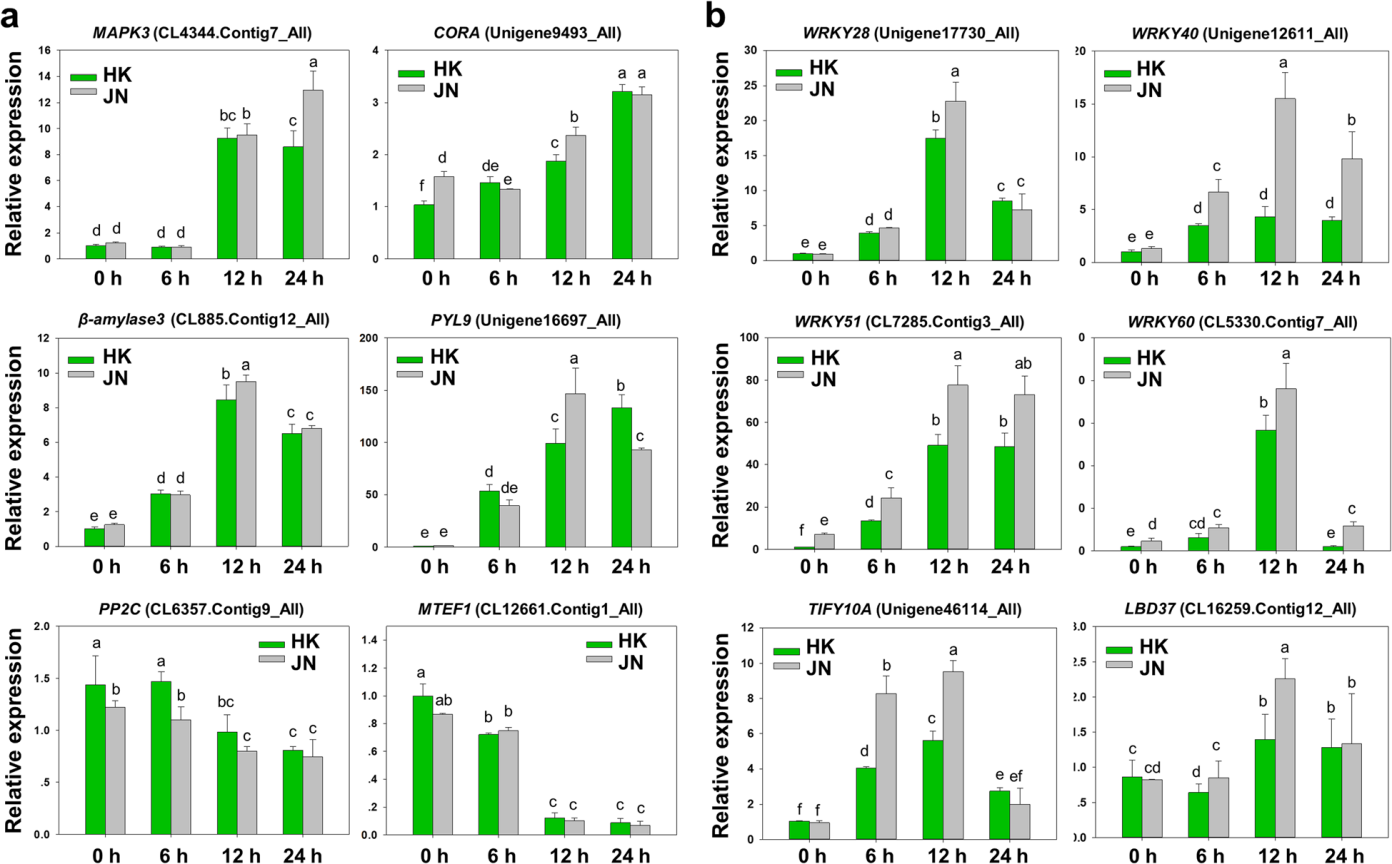
Expression of selected DEGs in response to cold stress by qRT-PCR. Data are the means±SD of three biological replicates. Bars with different letters are significantly different at *P* < 0.05 (ANOVA followed by Tukey’s post-hoc test)

To confirm the expression levels of the DEGs from the RNA-Seq data, twelve genes of HK and JN at different time points of cold treatment were selected for transcript abundance analysis (Fig. 9). Under cold stress, *MAPK3* (CL4344. Contig7_All), which increased at all time points compared to the control in the RNA-Seq analysis, exhibited a stable increase after 12 and 24 h of cold treatment. The expression of a cold and drought-regulated protein, *CORA* (Unigene9493_All), increased continuously and reached a significant increase at the 24 h time point. Similarly, β-amylase 3 (CL885. Contig12_All) and abscisic acid receptor *PYL9* (Unigene16697_All) were dramatically upregulated following cold treatment compared to the control levels. In contrast, ABA signalling *PP2C* (CL6357. Contig9_All) and transcription termination factor *MTEF1* (CL12661. Contig1_All) were downregulated following cold treatment compared to the control levels (Fig. 9a).

We then compared the expression levels of DEGs with significantly different expression profiles between JN and HK in the RNA-Seq data (Fig. 9b). Many transcription factors, such as those of the WRKY family, which is mainly involved in the key MAPK signalling pathway in cold stress, were elevated significantly in JN compared with HK. Four *WRKY* genes were selected for analysis under cold stress. *WRKY28* (Unigene17730_All), *WRKY40* (Unigene12611_All), *WRKY51* (CL7285. Contig3_All) and *WRKY60* (CL5330. Contig7_All) were more highly expressed in the cold-resistant JN plants than in the HN plants during cold treatment. Moreover, another jasmonate signalling repressor, *TIFY10A* (Unigene46114_All) and a *LATERAL ORGAN BOUNDARIES DOMAIN* (*LBD*) gene, *LBD37* (CL16259. Contig12_All), were upregulated in both JN and HN during the cold treatments, whereas the JN plants exhibited much higher expression levels than the HK plants, which was consistent with the DEG data. These results suggested that the RNA-Seq analysis was reliable.

### Epigenetic modification of the transcription factor genes in *A. philoxeroides* populations

**Fig. 10:**
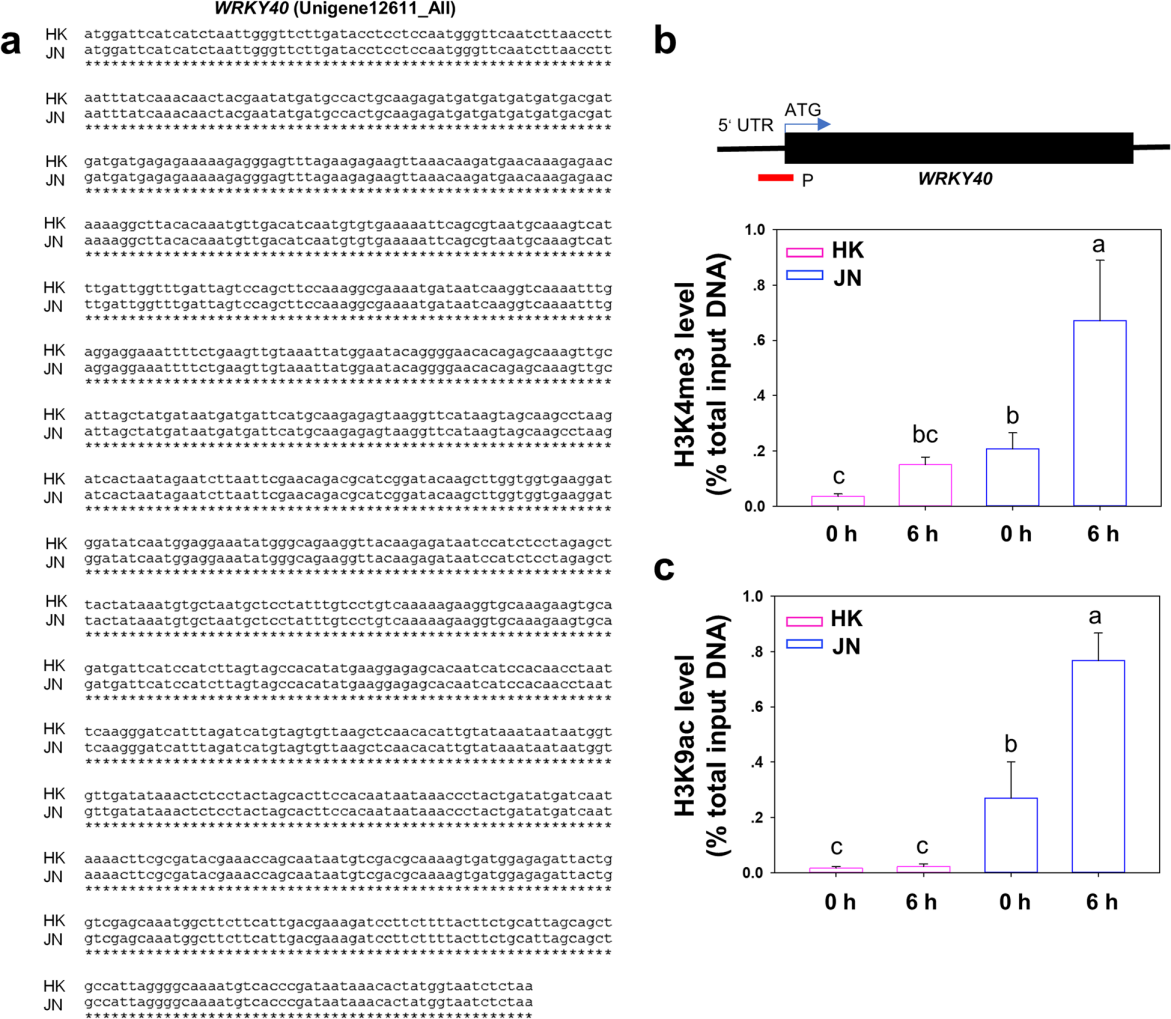
Histone epigenetic modification of the *WRKY40* gene in the two populations. **a**. Alignment of the nucleotide sequences of the coding region of the *WRKY40* gene in the HK and JN populations. Asterisks indicate the same nucleotide in these two populations. **b**. ChIP assay of the relative levels of H3K9ac and H3K4me3 in *WRKY40* chromatin of the HK and JN plants before and after cold treatment. The primers used in the PCR are shown as bars below the *WRKY40* gene. Data are the means±SD of triplicate experiments. Bars with different letters indicate significant differences at *P* < 0.05 (ANOVA followed by Tukey’s post-hoc test)

Previous studies showed that the alligator weed gradually expanded from south to north through asexual propagation, with a low level of DNA sequence variation [8]. Based on the expression profiles from the DEG data and qPCR validation analysis, many transcription factors, such as *WRKY* genes, *LBD* genes and *TIFY* genes exhibited significant variation at the transcription level in these two *A. philoxeroides* populations when exposed to cold. It is unlikely that genetic changes in gene sequences will occur in a short period of time, and epigenetic may play a role [21]. To test this possibility, we selected three candidate genes: *WRKY40* (Unigene12611_All), *TIFY10A* (Unigene46114_All) and *LBD37* (CL16259. Contig12_All) which increased prominently in the JN population compared to the HK population under cold stress, for epigenetic analysis. We first examined the full-length coding region of these genes in the JN and HK populations separately. Sequence alignments revealed that the nucleotide sequence of the *WRKY40* gene was exactly the same between the JN and HK populations (Fig. 10a). Previous studies showed that stress-induced genes that are upregulated following cold treatment were associated with the histone enrichment of H3K9 acetylation and H3K4 methylation (H3K9ac/H3K4me3), which are known as activating epigenetic marks, in the promoter region [21–23]. To investigate whether the histone modifications influenced the gene expression epigenetically between these two geographically distinct populations, we measured the levels of H3K9ac and H3K4me3 at the *WRKY40* gene locus before and after cold stress. Our results showed that cold treatment induced H3K4me3 levels at the transcriptional start site (TSS) of the first exon at the *WRKY40* genomic region increasing in both JN and HK plants, but H3K4me3 levels were higher in the JN population than in the HK population (Fig. 10b). Under the cold treatments, the JN plants also had higher levels of H3K9ac modification within the *WRKY40* locus than the HK plants (Fig. 10c). Furthermore, we noticed that the H3K4me3 levels and H3K9ac levels were different before cold stress, with higher levels in JN than in HK.

**Fig. 11:**
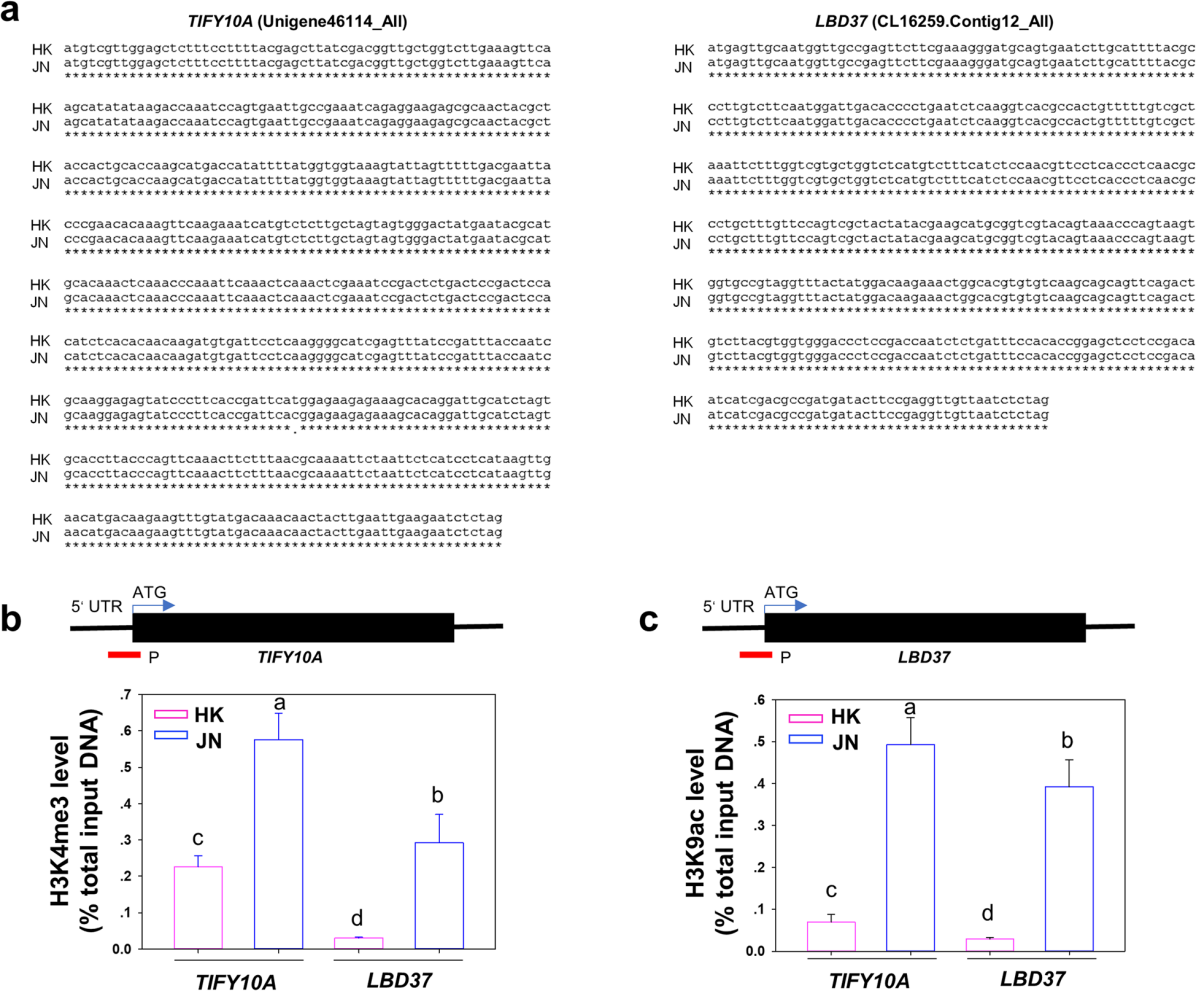
Histone epigenetic modification of the *TIFY10A* and *LBD37* genes in the two populations. **a**. Alignment of the nucleotide sequences of the coding region of the *TIFY 10A* and *LBD37* genes in the HK and JN populations. Asterisks indicate the same nucleotide in these two populations. **b**. ChIP assay of the relative levels of H3K9ac and H3K4me3 in *TIFY10A* and *LBD37* chromatins of the HK and JN plants before cold treatment. The primer used in the PCR is shown as bars below the genes. Data are the means±SD of triplicate experiments. Bars with different letters are significantly different at *P* < 0.05 (ANOVA followed by Tukey’s post-hoc test)

Many of stress-induced epigenetic changes can be stable and heritable through many generations through a molecular memory in plants [21]. We further tested the levels of H3K9ac and H3K4me3 of the *LBD37* and *TIFY10A* genes in the JN and HK populations collected from a local area (Fig. 11). We found that the levels of H3K9ac and H3K4me3 at the transcriptional start site (TSS) of these two gene loci were significantly higher in the JN population than in the HK population, even without cold treatments, with high nucleotide similarity of coding regions in these two genes (Fig. 11a-c). Together, these results indicated that variation in histone modifications of the three candidate genes primarily determines the transcript levels in response to cold stress, with no variation in sequence polymorphisms. This low temperature mediated potential histone epigenetic change could generate adaptive variation in *A. philoxeroides*.

## Discussion

*Alternanthera philoxeroides*, which first appeared in Shanghai (1930s) in China, was introduced to all parts of the country as pig feed in the early 1950s and later became a wild population in many areas. In fact, DNA molecular marker analysis showed that the genetic variation in the populations of alligator weed in China is very low, and these populations are probably the offspring of the same clone [8]. After invading the south of our country, alligator weed continued to expand northward and successfully invaded Shandong Province. In the tropics, alligator weed can grow and propagate all year round, but in many subtropical and temperate regions, the aboveground parts of this weed wither in early winter, and it mainly relies on the underground stems and storage roots for overwintering. Among the environmental factors that restrict its expansion, the continuous decrease in temperature from south to north is very important. To successfully invade northern China and survive the winter, it is necessary for this species to increase its cold resistance. In this study, our results showed that the JN population had more freezing tolerance than the HK population. The HK population was very sensitive to freezing temperatures, suggesting a close relationship between geographical distribution and cold tolerance (Figs. 2, 3). Previous studies have found that low temperature limits the geographical distribution, and it is also a key environmental factor controlling the northward advance of invasive species [12, 24].

To tolerate cold stress, plants have evolved various effective mechanisms [25]. One of the key mechanisms is cold acclimation. In this process non-freezing cold stress can induce cold adaptation in plants to improve the freezing resistance of plants [26]. In this study, to investigate the cold resistance mechanism of alligator weed and the difference in this mechanism after spreading from south to north, a comparative transcriptome analysis of two different populations from Haikou city and Jinan city of China under cold stress was performed. Differential expression analyses revealed that most DEGs were present in the HK and JN populations during cold stress. Many DEGs were induced by cold temperature, and more upregulated unigenes were identified than downregulated unigenes at all time points of cold stress (Fig. 4). GO enrichment and KEGG pathway analysis showed that protein kinase-related genes, transcription factors, plant-pathogen interactions, plant hormone signal transduction and metabolic processes might be significant in the alligator weed response to low temperature (Fig. 8).

At the beginning of cold stress, rapid Ca^2+^ signalling and mitogen-activated protein kinase (MAPK) cascades are activated. A series of calcium decoding proteins were dramatically activated in both *A. philoxeroides* populations, including calmodulin-like protein (Unigene26948_All), calcium-dependent protein kinase (Unigene11118_All, Unigene20452_All), calcium B-like protein (CL2709. Contig11_All, CL2709. Contig20_All), calcium-transporting ATPase (Unigene36447_All, Unigene36450_All) and calmodulin-binding proteins (CL1143. Contig21_All, Unigene10926_All, Unigene17219_All). These calcium related proteins play key roles as Ca^2+^ sensors and activities downstream in response to cold stress [27, 28]. MAPK cascades also have important functions in cold responses. Our results showed that three typical protein kinases, MAP kinase kinase kinase (MAPKKK), MAP kinase kinase (MAPKK) and MAP kinase (MAPK), were activated during cold stress. Transcription factors are important regulators in response to cold stress [29]. CBFs/DREB transcription factors bind to the cis-elements of cold-responsive genes (CORs) and upregulate their expression to enhance cold resistance in plants [30]. We found that the cold-induced *CORA* gene (Unigene9493_All) was identified and increased in both the JN and HK populations. Numerous TF families, such as *WRKYs*, *NACs* and *MYBs* were induced by cold stress in *A. philoxeroides*. Some of these transcription factors are reported to mediate the *COR* genes expression in response to cold stress [31]. Our results showed that *WRKY40* (Unigene12611_All), *WRKY51* (CL7285. Contig3_All) and *WRKY60* (CL5330. Contig7_All) were activated in both the JN and HK populations, especially in JN, with higher expression than that in HK. *WRKYs* are activated by MAPK cascades, and modulate *COR* genes independent of CBFs in a temperature-dependent manner during chilling stimulus [32, 33]. *HSFA1* (*Heat stress transcription factor 1*, Unigene5244_All) expression was found to increase during cold treatment. Recently, *HSFA1* was reported to positively regulate the cold tolerance of plants [34]. Plants also integrate hormone and cold signalling pathways to improve their adaptation to cold stress [35–37]. During the cold treatment process, various hormone signalling pathways were also involved in the cold tolerance of alligator weed, including jasmonate signalling, abscisic acid signalling, and ethylene and auxin signalling. Apart from the above signal regulators, we also found that the rapid accumulation of soluble sugars, proline and other small molecules, such as polyamine, played protective roles in the cold stress response. These results will help to demonstrate that low temperature is the key environmental factor limiting the expansion of *Alternanthera philoxeroides*, and to understand the physiological response and molecular mechanism of cold tolerance divergence of this seed under low temperature stress.

Although many invasive alien species can increase their genetic diversity through sexual reproduction and hybridisation to continuously adapt to the new environment, some species that rely on expansion via asexual reproduction and that have low genetic diversity need to expand and adapt to the new environment through epigenetic variation to maintain ecological phenotypic variation and plasticity [38]. Epigenetic regulation plays key roles in plant cold tolerance [39]. Plants exposed to cold stress-like conditions induced increased H3K4me3 levels at the genomic level, which are associated with the transcriptionally active loci of stress-responsive genes [40]. In this study, our results showed that many cold signal transduction genes were more highly upregulated in JN than in HK in response to chilling stimuli. Further results showed that the levels of histone H3K9 acetylation and H3K4 methylation (H3K4me3/H3K9ac) at three candidate gene loci were higher in the JN population than in the HK population before and after the cold treatments, resulting in higher expression of these genes in the JN plants than in the HK plants. These results suggest that the northern population maintains more histone H3K4me3 and H3K9ac enrichment than the southern population, especially before cold treatments (Figs. 10, 11).

Emerging evidence has indicated that epigenetic variation in plant populations can be an important mechanism in plant invasions and has key roles in rapid adjustment of plants to the environment [41, 42]. In this study, epigenetic variation of the southernmost and the northernmost populations of alligator weed may be associated with the adaptive evolution of increased tolerance to cold stresses with physiological plasticity. Our previous study also showed that the genetic diversity in the introduced ranges was lower and that the phenotypic plasticity was higher than in the native range [8]. This study will help us to understand the strategies for northward invasion of alligator weed, and to better anticipate the potential adaptation and expansion range of *A. philoxeroides*. Alternatively, cold-resistance changes could also involve DNA methylation variation at cold-responsive gene loci that influence gene expression [43]. Further study will focus on the genome-wide pattern of DNA methylation divergence and stability in the transplanted seedlings between these two populations.

## Conclusions

Low temperature is the key limiting environmental factor for the expansion of alligator weed from the south to the north in China. In the present study, we comprehensively described the difference in cold tolerance between the southernmost population and the northernmost population at the transcriptional level. Different expression levels of cold stress-responsive genes between these two populations belonged to the major pathways associated with protein kinases involved in signal transduction, transcription factors, plant hormones, and metabolite biosynthesis groups. Many cold-responsive genes exhibited much higher expression levels in the JN population than in the HK population in the same signal pathway, especially transcription factors. Histone epigenetic modification experiments showed that the candidate genes in JN plants had higher levels of H3K4me3 and H3K9ac enrichment at the related gene locus, which was consistent with the high levels of gene expression in JN under cold stress. However, there were no differences in the coding sequences of these genes in these two populations. These results indicate that epigenetic modification may influence cold resistance during the invasion of alligator weed from south to north. Epigenetic regulation has the potential to maintain information over time for environmental stimuli and rapidly introduce heritable phenotypic effects with memory [44]. Our study provides direct evidence to link the histone modifications in cold-responsive genes with the genetic variation and cold adaptation underlying the northward expansion of alligator weed.

## Methods

### Plant materials and stress treatments

The samples belonging to the two populations were simultaneously collected from Hainan Province (Jinan City, 36°26′N, 117°27′E) and Shandong Province of China (Haikou City, 20°02′N, 110°11′E) in June 2018. These materials were identified by Professor Yupeng Geng, an expert who had studied this species for more than 20 years. The voucher specimens (No. YNU2014078 and YNU 2014079) of these materials were deposited in the herbarium of Yunnan University. For each individual, a stem fragment was transplanted in a pot with soil and vermiculite at a 3:1 ratio in an greenhouse at 22 °C under long-day (LD) conditions (16-h light/8-h dark photoperiod). The freezing and chilling tests of the two *A. philoxeroides* populations were further performed using the transplanted seedlings. For the freezing treatment, one-month-old transplanted plants were placed in a freezing chamber that was set to − 1 °C, and the temperatures decreased to − 4 °C or − 5 °C. After the treatment, plants were incubated at 4 °C overnight and then placed in greenhouse. For the chilling treatment, the plants were placed in an incubator at 4 °C under short-day (SD) conditions (8-h light/16-h dark) for 6, 12 or 24 h. The survival rates and relative electrolyte leakage levels of the seedlings were calculated as previously described [45]. Chl fluorescence was measured using an IMAGING-PAM Chl fluorimeter, and the *Fv*/*Fm* ratios were calculated using Imaging WinGegE software (Walz, Germany). All measurements were repeated in three independent experiments. Data are expressed as the means±standard deviation (SD) of three biological replicates.

### RNA isolation and sequencing

Leaves of eighteen samples with each 2 g from the HK and JN seedlings with and without cold treatment were sent to BGI Co. (Shenzhen, China) for RNA isolation and sequencing. Briefly, total RNA was extracted using an EASY-spin plant RNA Extraction Kit (Aidlab China) following the manufacturer’s instructions. RNA was treated by mRNA enrichment and rRNA removal, and the required RNA was obtained after purification. The obtained RNA was segmented with interrupt buffer and transcribed with random N6 primers, and then the two strands of cDNA were synthesised to form double-stranded DNA. The double-stranded cDNAs were then end-repaired and phosphorylated, and a polyA tail was added following Illumina’s library construction protocol. The fragments were selected via agarose gel electrophoresis and amplified using Phusion DNA polymerase (NEB). PCR products were heat denatured into a single strand, and then a single strand DNA library was obtained by cyclisation of single strand DNA with a bridge primer. The Illumina BGISeq-500 platform was used for DNA library sequencing.

### De novo assembly and gene annotation

The raw reads were filtered, removing the reads with low quality (*Q-value* < 20) and the reads with a high content of ambiguous “N” bases (more than10%), and clean reads with high quality were obtained. The clean reads were employed for de novo assembly using Trinity software (http://trinityrnaseq.github.io) [46], and then the transcripts were clustered and further assembled according to paired-end information to obtain the unigenes. BUSCO software (https://busco.ezlab.org/) was used to evaluate the quality of the assembled transcripts, and TransDecoder software (https://github.com/TransDecoder/TransDecoder) was used to identify the candidate coding regions of the unigenes. The assembled unigenes were annotated with seven functional databases, including KEGG (Kyoto Encyclopedia of Genes and Genomes) (http://www.genome.jp/kegg), GO (Gene Ontology) (http://geneontology.org), NR (NCBI non-redundant protein database) (ftp://ftp.ncbi.nlm.nih.gov/blast/db), NT (NCBI nucleotide databases) (ftp://ftp.ncbi.nlm.nih.gov/blast/db), Swiss-Prot, Pfam (http://pfam.xfam.org) and COG (Clusters of Orthologous Groups) (https://www.ncbi.nlm.nih.gov/COG/).

### Analysis and functional enrichment of DEGs

Bowtie2 software was used to match clean reads to reference genome sequences, and then RSEM (http://deweylab.biostat.wisc.edu/rsem/) [47] was used to calculate the gene expression level of each sample to obtain the FPKM (fragments per kilobase of exon per million mapped fragments) values of each gene. Based on these statistical analyses, The resulting *P* values were corrected to *Q* value for controlling the false discovery rate (FDR). Genes with an adjusted *Q* < 0.05 calculated by DESeq (v1.18.0) [48] and an absolute value |Log_2_(fold-change)| ≥ 4 (FC) were considered to be significantly differentially expressed genes (DEGs). GO enrichment analyses of DEGs were obtained using Goatools (https://github.com/tanghaibao/Goatools) and KOBAS (http://kobas.cbi.pku.edu.cn/expression.php) with GO annotation [49, 50]., and FDR correction was carried out for *Q*-value analysis. A *Q*-value≤0.05 was the threshold for significant enrichment. DEGs were subjected to KEGG pathway annotation and classified into different biological pathways, KEGG pathway enrichment analysis was also implemented using the same method. FDR correction was used to test the *Q value*, and a *Q*-value ≤0.05 was the threshold for significant enrichment.

### Quantitative real-time PCR

Quantitative real-time PCR analysis was performed as reported previously [51]. Total RNA from the leaves of HK or JN plants was extracted using TRIzol reagent (Invitrogen) before and after the cold treatments. First-strand cDNA was synthesised from 1.5 μg of DNase-treated RNA in a 20 μL reaction volume using M-MuLV reverse transcriptase (Invitrogen) with an oligo (dT)_18_ primer. qRT-PCR was conducted using 2 × SYBR Green I Master on a Roche LightCycler 480 real-time PCR machine following the manufacturer’s instructions. At least three biological replicates and three technical replicates for each sample were used in the qRT-PCR analysis. The gene-specific primers used to detect transcripts are listed in Additional file 18: Table S16.

### Cloning and sequence analysis

To verify the low level of genetic variation in the JN and HK populations, the full-length cDNA coding regions of the three candidate genes *WRKY40*, *LBD37* and *TIFY10A* from JN and HK plants were amplified according to the RNA-Seq data. Gene-specific primer sequences are listed in Additional file 18: Table S16. Sequence alignments of the deduced amino acids were performed using MAFFT software (https://mafft.cbrc.jp/alignment/software/) [52].

### ChIP assay

ChIP was performed largely as described previously [53]. For each assay, ~ 0.6 g of the leaves from HK or JN plants were harvested before and after the cold treatments and were cross-linked by 37% formaldehyde in phosphate buffered saline (PBS) buffer. Further ChIP assays were performed using a Magna ChIP™ A-Chromatin Immunoprecipitation Kit (Millipore) following the manufacturer’s instructions. Immunoprecipitation was performed with acetylated H3 (11,000; Millipore), acetyl H3K9 and trimethyl H3K4 (1500; Millipore) antibodies. Both immunoprecipitated DNA and input DNA were analysed by real-time PCR. Normalisation of the immunoprecipitated DNA amplification over the input amplification gives a reliable readout of the ChIP efficiency. Primers were as described (Additional file 18: Table S16). Data from the ChIP assay are expressed as the means±standard deviation (SD) of three biological replicates.

## Supplementary information

**Additional file 1: Figure S1.** Distribution of unigene lengths in *A. philoxeroides* transcriptome. **Figure S2.** Quality assessment of assembled transcripts using BUSCO analysis. **Figure S3.** Principal component analysis (PCA) factorial maps showing the largest components of variance. **Figure S4.** Annotation of unigenes of *A. philoxeroides* by NR database. **Figure S5.** Comparison of the KEGG pathway annotation for DEGs in HK (a) and JN (b) (*Q*-value≤0.05). The X-axis is the number of genes annotated to a certain KEGG pathway category, and the Y-axis is the KEGG pathway. **Figure S6.** KEGG enrichment analysis of the common and unique DEGs in HK and JN populations. **a.** Venn diagram analysis showing the common and unique DEGs in HK and JN. **b-d.** KEGG enrichment analysis of the common DEGs in both HK and JN (b), unique DEGs in HK (c) and unique DEGs in JN (d) (*Q*-value≤0.05). **Figure S7.** KEGG enrichment analysis during the two cold treatment time points in HK and JN populations. **a.** KEGG enrichment analysis of HK-12 h. **b.** KEGG enrichment analysis of HK-24 h. **c.** KEGG enrichment analysis of JN-12 h. (*Q*-value≤0.05). **d.** KEGG enrichment analysis of JN-24 h. The top pathways with minimum *Q values* (*Q*-value≤0.05) are shown.

**Additional file 2: Table S1.** Statistics of sequencing reads from the HK and JN populations with different low-temperature treatments.

**Additional file 3: Protein.fa.** Predicted protein sequence.

**Additional file 4: Table S2.** Summary statistics for the comprehensive transcriptome of *A. philoxeroides*.

**Additional file 5: Table S3.** BUSCO analysis for quality assessment of assembled transcripts.

**Additional file 6: Table S4.** FPKM values for functional DEGs in the HK and JN populations with different low-temperature treatments.

**Additional file 7: Table S5.** List of the expression patterns of DEGs in HK and JN under cold stress.

**Additional file 8: Table S6.** Expression pattern of DEGs between the HK and JN populations under cold treatments.

**Additional file 9: Table S7.** GO annotation analysis for the total number of DEGs.

**Additional file 10: Table S8.** GO enrichment analysis for the total number of DEGs.

**Additional file 11: Table S9.** KEGG pathway annotation analysis for the total number of DEGs.

**Additional file 12: Table S10.** KEGG enrichment analysis for the total number of DEGs.

**Additional file 13: Table S11.** KEGG enrichment analysis for DEGs during the two cold treatment time points in HK and JN populations.

**Additional file 14: Table S12.** DEGs related to protein kinases in the HK and JN populations.

**Additional file 15: Table S13.** DEGs related to transcription factors in the HK and JN populations.

**Additional file 16: Table S14.** DEGs related to plant hormones in the HK and JN populations.

**Additional file 17: Table S15.** DEGs related to metabolite biosynthesis in the HK and JN populations.

**Additional file 18: Table S16.** Primers used for qRT-PCR and ChIP analysis.

## Data Availability

All data generated in this study are included in the main article and its supplementary files. All the transcriptome data have been deposited in the NCBI’s Sequence Read Archive (SRA) with accession No. PRJNA599518 (Available online: https://www.ncbi.nlm.nih.gov/sra/PRJNA599518). This Transcriptome Shotgun Assembly project has been deposited at DDBJ/EMBL/GenBank under the accession No. GISA00000000 in the FASTA format.

## Abbreviations

LD: Long-day
SD: Short-day
DEGs: Differentially expressed genes
PCA: Principal component analysis
NR: NCBI non-redundant protein database
NT: NCBI nucleotide databases
COG: Clusters of Orthologous Groups
FPKM: Fragments per kilobase of exon per million mapped fragments
FC: Fold change
KEGG: Kyoto Encyclopedia of Genes and Genomes
GO: Gene Ontology
MAPK: Mitogen-activated protein kinase
RLKs: Receptor-like kinases
ABA: Abscisic acid
*PP2C*: ABA co-receptor phosphatase 2C
*LBD*: Lateral organ boundaries domain
qRT-PCR: Quantitative real-time PCR
ChIP: Chromatin Immunoprecipitation
TSS: Transcriptional start site
H3K4me3: H3K4 methylation
H3K9ac: H3K9 acetylation
