# Supplementary figures and images for: Comparative transcriptome analysis reveals ecological adaption of cold tolerance in northward invasion of Alternanthera philoxeroides
Source: BMC Genomics. 2020 Aug 2;21:532. doi: 10.1186/s12864-020-06941-z (PMC7430914; doi:10.1186/s12864-020-06941-z)

Fig. S1

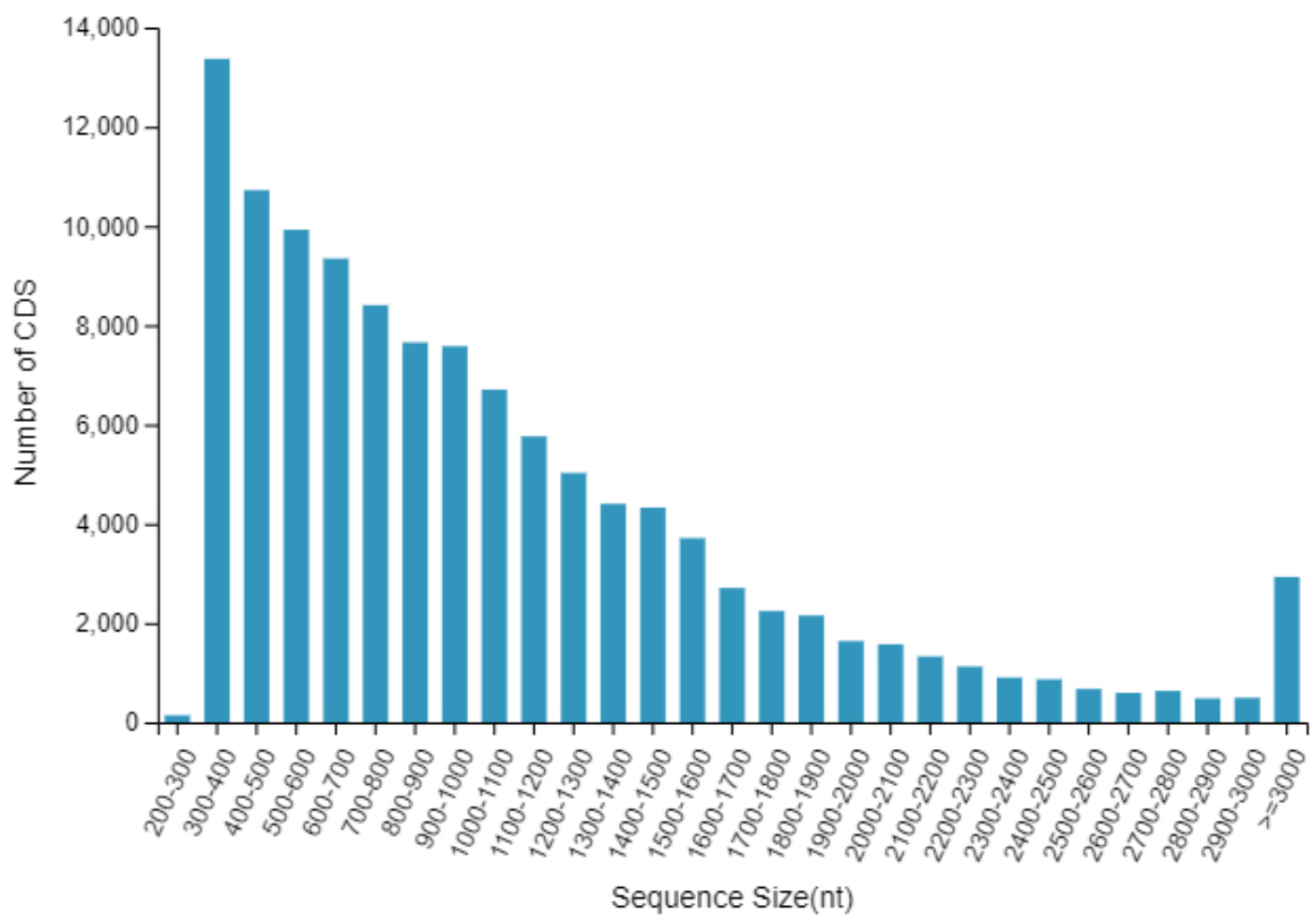

Fig. S2

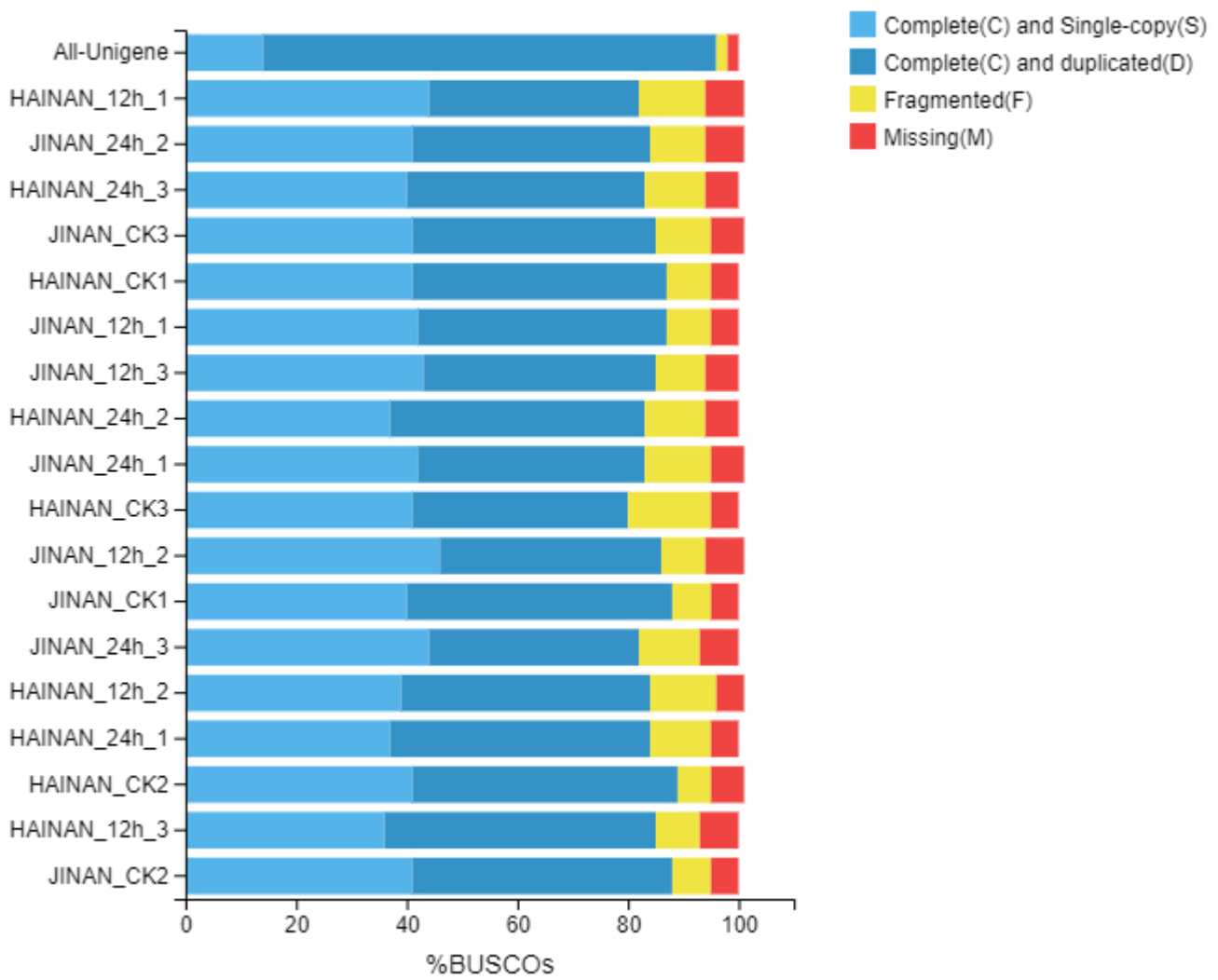

Fig. S3

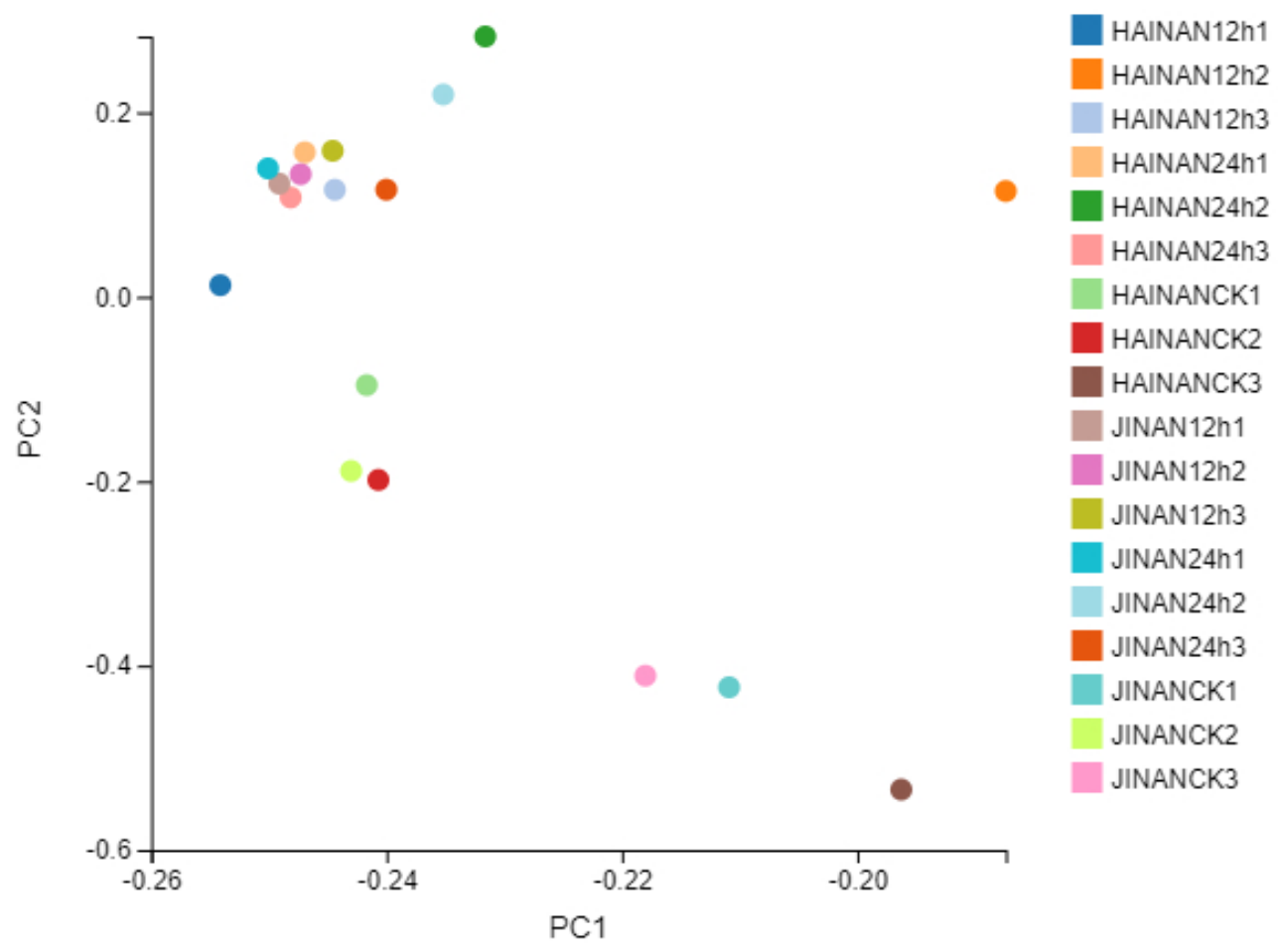

Fig. S4

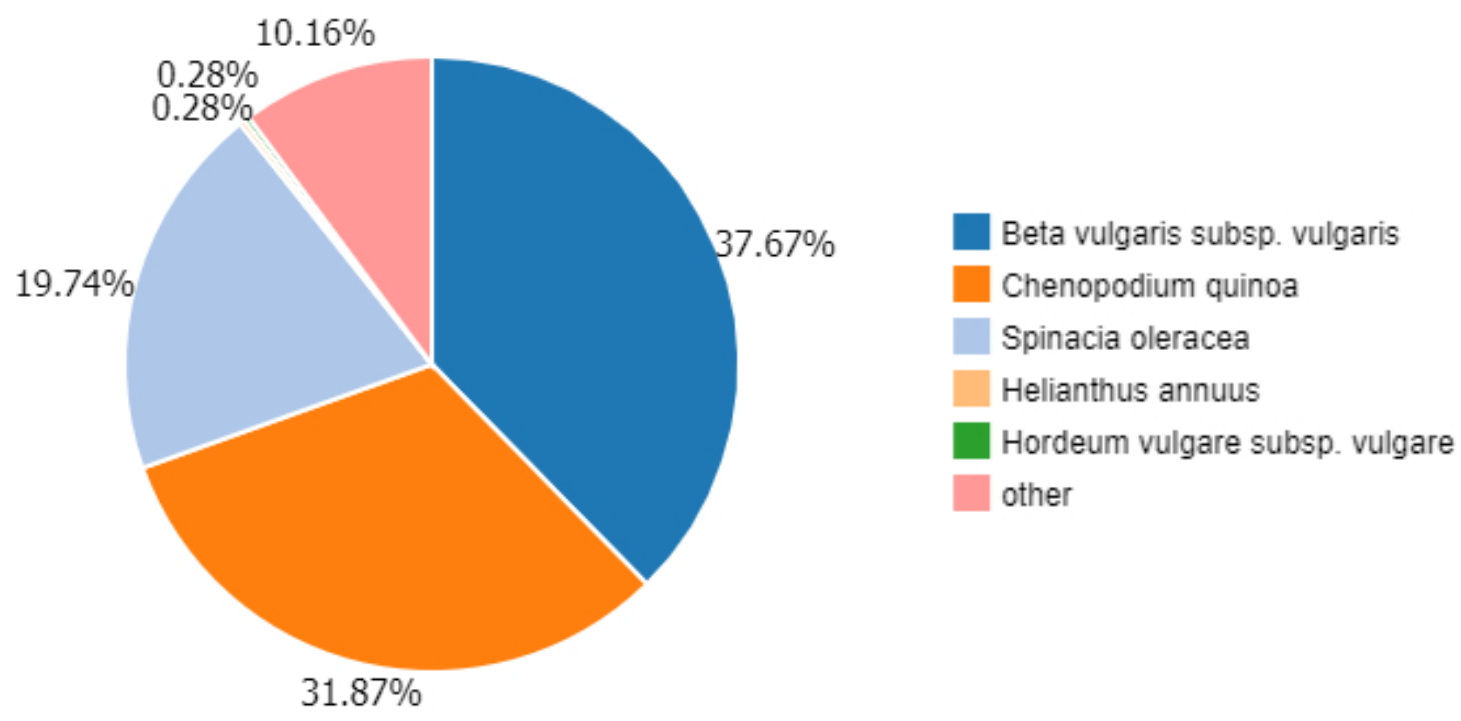

Fig. S5

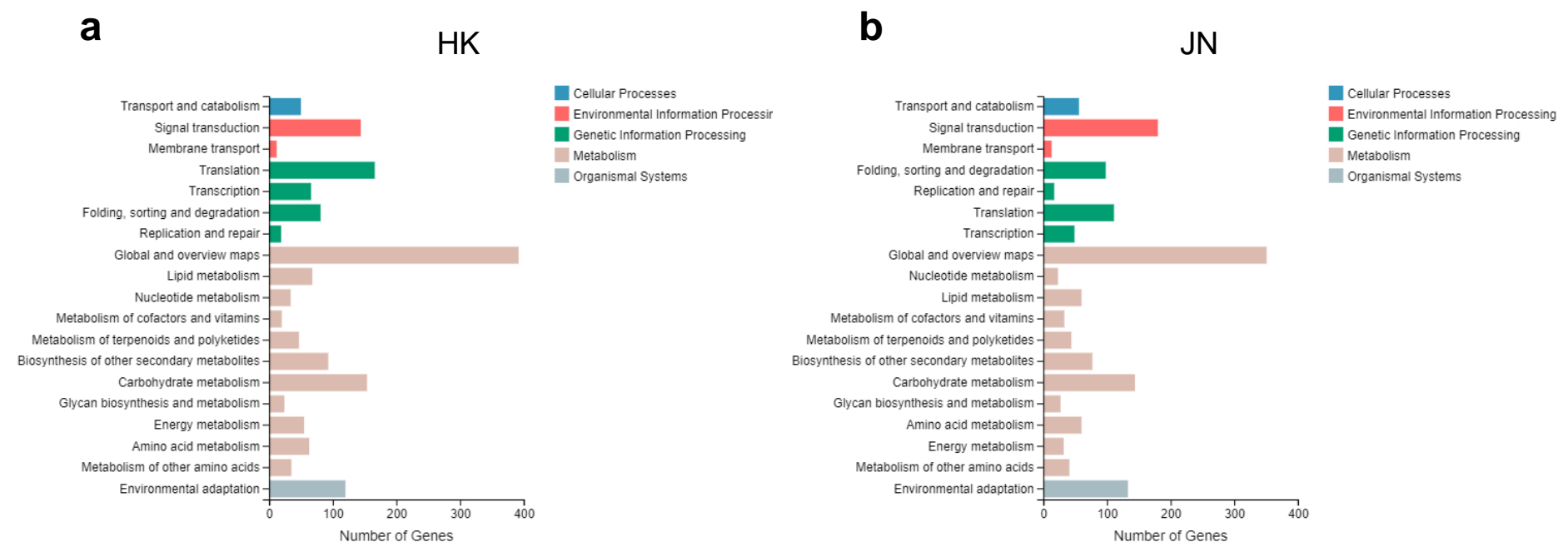

Fig. S6

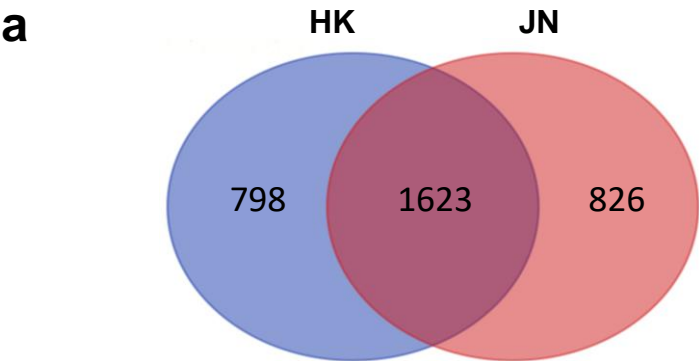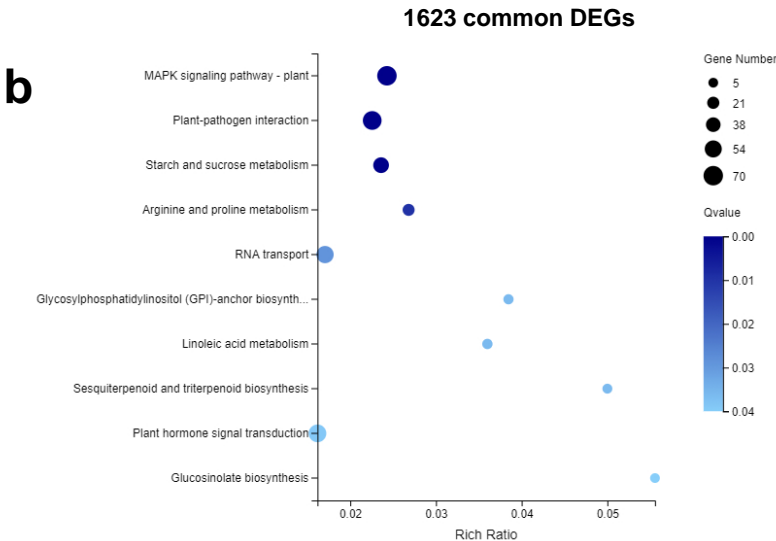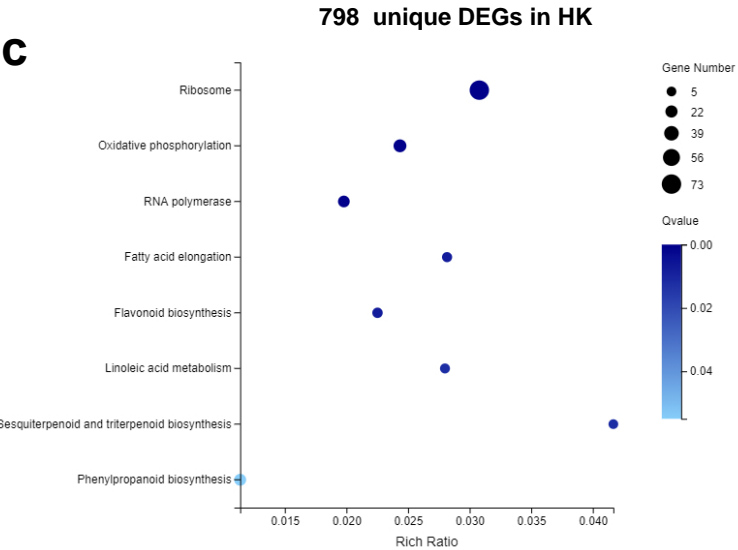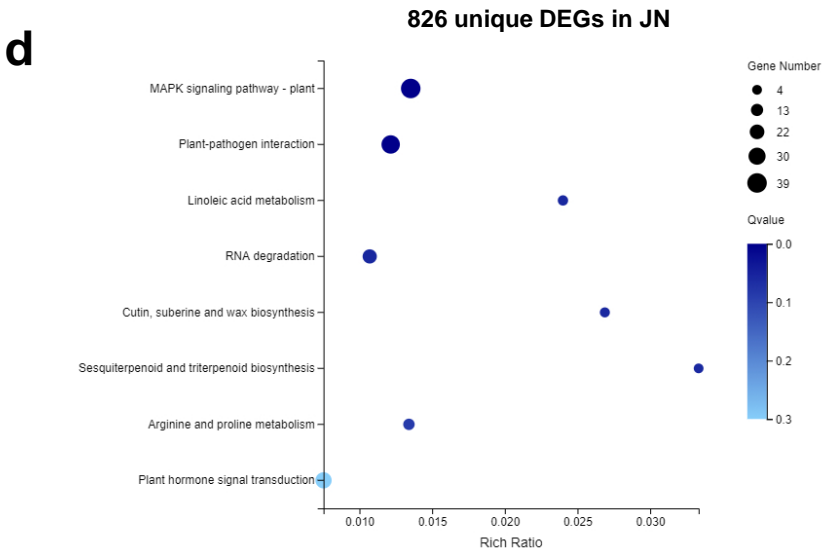

Fig. S7

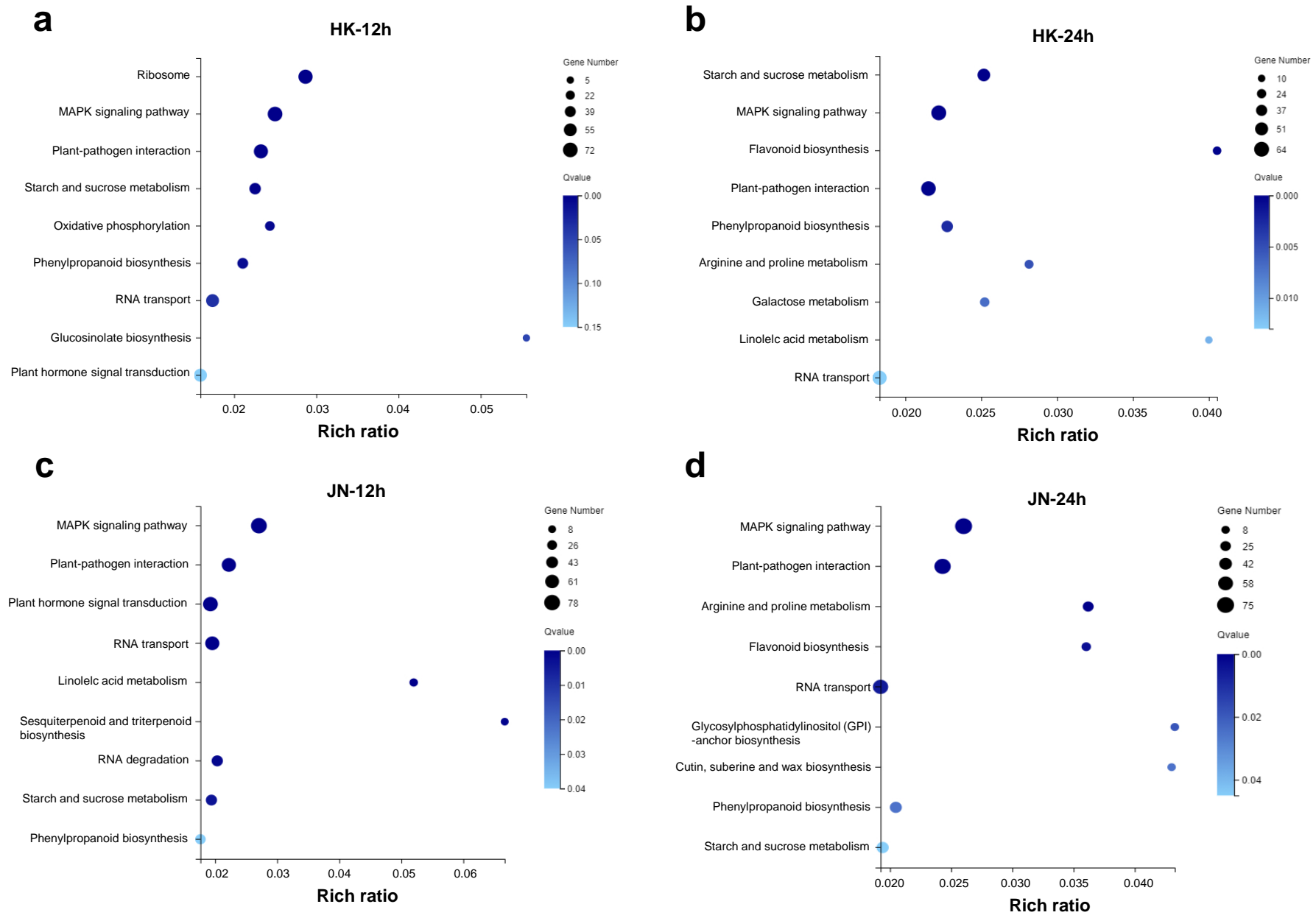

Supplement: Supplementary file 1 — Additional file 1: Figure S1. Distribution of unigene lengths in A. philoxeroides transcriptome. Figure S2. Quality assessment of assembled transcripts using BUSCO analysis. Figure S3. Principal component analysis (PCA) factorial maps showing the largest components of variance. Figure S4. Annotation of unigenes of A. philoxeroides by NR database. Figure S5. Comparison of the KEGG pathway annotation for DEGs in HK (a) and JN (b) (Q-value≤0.05). The X-axis is the number of genes annotated to a certain KEGG pathway category, and the Y-axis is the KEGG pathway. Figure S6. KEGG enrichment analysis of the common and unique DEGs in HK and JN populations. a. Venn diagram analysis showing the common and unique DEGs in HK and JN. b-d. KEGG enrichment analysis of the common DEGs in both HK and JN (b), unique DEGs in HK (c) and unique DEGs in JN (d) (Q-value≤0.05). Figure S7. KEGG enrichment analysis during the two cold treatment time points in HK and JN populations. a. KEGG enrichment analysis of HK-12 h. b. KEGG enrichment analysis of HK-24 h. c. KEGG enrichment analysis of JN-12 h. (Q-value≤0.05). d. KEGG enrichment analysis of JN-24 h. The top pathways with minimum Q values (Q-value≤0.05) are shown. [file 12864_2020_6941_MOESM1_ESM.pdf]
